# Integrated prediction of lung cancer histology and molecular profiles from small bronchoscopic tumor specimens

**DOI:** 10.1038/s41698-026-01623-7

**Published:** 2026-07-27

**Authors:** Marija Karadzovska-Kotevska, Anna Karlsson, Hans Brunnström, Mats Jönsson, Jaroslaw Kosieradzki, Stefan Barath, Anders Näslund, Christel Estberg, Elsa Arbajian, Frida Rosengren, Srinivas Veerla, Janne Lehtiö, Lukas M. Orre, Johan Staaf, Maria Planck

**Affiliations:** 1 Department of Clinical Sciences Lund, Division of Oncology, Lund University, Lund, Sweden; 2 Department of Laboratory Medicine, Division of Translational Cancer Research, Lund University, Lund, Sweden; 3 Department of Respiratory Diseases and Allergology, Skåne University Hospital, Lund, Sweden; 4 Department of Clinical Sciences Lund, Division of Pathology, Lund University, Lund, Sweden; 5 Department of Oncology and Pathology, Karolinska Institute, Science for Life Laboratory, Solna, Sweden; 6 Department of Clinical Sciences Lund, Division of Respiratory Medicine, Allergology, and Palliative Medicine, Lund University, Lund, Sweden

**Keywords:** Biomarkers, Cancer, Oncology

## Abstract

Treatment decisions in lung cancer rely on comprehensive molecular and histological characterization, but the limited amount of tumor tissue available from diagnostic procedures remains a major challenge. In this proof-of-concept study, we investigated whether preserving small bronchoscopic tumor specimens in RNAlater enables integrated molecular and histological profiling. Bronchial forceps biopsies and endobronchial ultrasound-guided transbronchial needle aspiration (EBUS-TBNA) specimens were collected during the diagnostic work-up of patients with suspected lung cancer, preserved in RNAlater, and used for DNA, RNA, and protein extraction. RNA was analyzed using NanoString gene expression profiling and RNA-sequencing to assess treatment-relevant gene fusions, *MET* exon 14 skipping events, and histological subtypes. Somatic mutations were identified by massively parallel sequencing, and proteomics-based non-small cell lung cancer (NSCLC) subtypes were characterized by mass spectrometry. High-quality DNA, RNA, and proteins were successfully recovered from all tumors. Gene fusion analysis was successful in 44 of 45 cases. Gene expression-based histological classification showed good concordance with the clinical pathology diagnosis, although some discrepancies were observed. Mutation profiling was fully concordant with routine clinical testing, and comprehensive proteomic data were successfully generated. Collectively, these findings demonstrate that RNAlater-preserved bronchoscopic specimens provide material of sufficient quality for integrated multi-omics analyses, enabling comprehensive molecular profiling from a single, small tumor biopsy.

## Introduction

Lung cancer is the most commonly diagnosed cancer and is responsible for the highest number of cancer-related deaths^1^. New treatment alternatives, targeting tumor-specific mutations and gene fusions, require simultaneous morphologic, immunohistochemical (IHC), and comprehensive genomic and transcriptomic profiling of the tumor to guide clinical decisions. The National Comprehensive Cancer Network (NCCN) and the International Association for the Study of Lung Cancer (IASLC) both recommend concurrent diagnosis, staging, and molecular genetic testing during the primary investigation of patients with suspected lung cancer^2,3^. Endoscopic sampling techniques, such as bronchoscopy with endobronchial ultrasound-guided transbronchial needle aspiration (EBUS-TBNA), play a critical role in the diagnostic work-up of lung cancer^4^. These minimally invasive procedures are characterized by high diagnostic accuracy and a low complication rate^5,6^.

Endoscopically acquired tumor biopsy specimens are typically formalin-fixed and paraffin-embedded (FFPE) to allow morphological and immunohistochemical evaluation. The FFPE preservation process can, however, induce crosslinking, fragmentation and base modification leading to sequence errors, with RNA being particularly susceptible to degradation^7–9^. These alterations compromise the quality of extracted nucleic acids and may result in inconclusive results from treatment-predictive DNA- and RNA-based molecular analyses. In contrast, molecular profiling of lung cancer for research purposes, using genome-wide genomics, transcriptomics, epigenomics and proteomics, has typically relied on fresh-frozen tumor tissue as the primary source of intact nucleic acids and proteins^10–14^. This approach requires stringent tissue handling conditions, including minimizing the time from sampling to low-temperature storage, and the selection of tumors available for molecular analysis has been largely limited to surgically resectable early-stage disease. To address the problem of nucleic acid degradation, especially RNA instability, preservative solutions such as RNAlater have been developed and have enabled large-scale RNA-sequencing, for example, in early-stage breast cancer in a routine clinical context^15,16^. Moreover, in lung cancer, we have shown that preservation of tissue in RNAlater is a sufficient source for protein extraction, allowing for in-depth proteomics using mass spectrometry^17^.

In this study, we aimed to assess whether preservation of small bronchoscopic specimens, such as bronchial forceps biopsies and EBUS-TBNA aspirates, in RNAlater is a feasible approach for the integrated prediction of molecular and histological characteristics of lung cancer. Specifically, we investigated whether RNAlater-preserved bronchoscopic specimens could, as a single-sample source, support proteomic analysis, RNA-based prediction of lung cancer histology, and detection of treatment-predictive gene fusions, splice variants, and gene mutations. Importantly, improved specimen management procedures may enhance the value of biobank collections from patients with non-resectable late-stage lung cancer by providing higher-quality material for both diagnostic and research purposes, which will also increase future possibilities of using more comprehensive applications.

## Results

### Fusion gene status and METex14 skipping events

The fusion gene status of *ALK*, *RET*, *ROS1*, *NRG1*, and *NTRK1*, as well as *MET*ex14 skipping events, was assessed for the 44 samples that met the quality control standards set by NanoString and compared to data from routine clinical analysis. One canonical *EML4::ALK* fusion (*EML4* exon 6::*ALK* exon 20) detected in routine clinical testing (by the Oncomine Focus NGS panel on FFPE RNA) could neither be confirmed by NanoString analysis nor by RNAseq using the FusionCatcher tool. One *MET*ex14 skipping event was detected by NanoString that had not been reported in the routine clinical testing (based on Oncomine Focus data).

### Mutation detection

To assess the feasibility of RNAlater-preserved specimens for mutation detection, we compared targeted NGS results using the Illumina TST-15 panel to reported mutations from treatment-predictive mutation screening performed in clinical routine, finding 100% concordance (Table 1).

**Table 1:** Gene expression-based histology prediction, proteomic classification and mutation detection

		PATHOLOGY department review				
		AC	NOS		SCLC	SqCC
SSP prediction on NanoString-derived gene expression data *n* = 44	**AC**	7	2		0	1
	**NE**	2	1		8	0
	**SqCC**	10	1		0	12
SSP prediction on RNAseq derived gene expression data *n* = 23	**AC**	4	1		0	0
	**NE**	1	1		2	0
	**SqCC**	7	0		1	6
Proteomic classification *n* = 44	**AC enriched**^**a**^	13	0		1	2
	**NE**	0	0		5	0
	**SqCC**	4	0		0	7
	**Unclassified**	1	0		0	1
	**Not classified**	3	2		2	3
Gene alteration identified by mutation detection using NGS		**Patients identified in clinical routine** (***N***)		**Patients identified in the current study** (***N***)		**Concordance**
***EGFR***		5		5		100%
***KRAS***		4		4		100%
***PIK3CA***		4		4		100%

An SSP capable of NSCLC histology prediction was applied to gene expression data derived by NanoString and RNAseq. Proteomic classification was extracted from previously generated data^17^. Mutation detection was performed using the Illumina TST-15 NGS platform.

^a^Proteomics class ST1, ST2, ST3.

### Histology prediction using gene expression data

A previously reported gene expression-based SSP of NSCLC histology was applied to the non-normalized gene expression data generated by NanoString (*n* = 44) and RNAseq (*n* = 23)^18^. Predicted histology was compared with histology assessment in clinical routine (Table 1). The accuracy of the SSP compared with the routine histological assessment was 0.61, with a balanced accuracy of 0.75. The SSP was also applied to gene expression data generated by RNAseq, with an accuracy of 0.57 and balanced accuracy of 0.74 compared to the routine assessment (Table 1).

### Histology review of discrepant histological cases

**Fig. 1:**
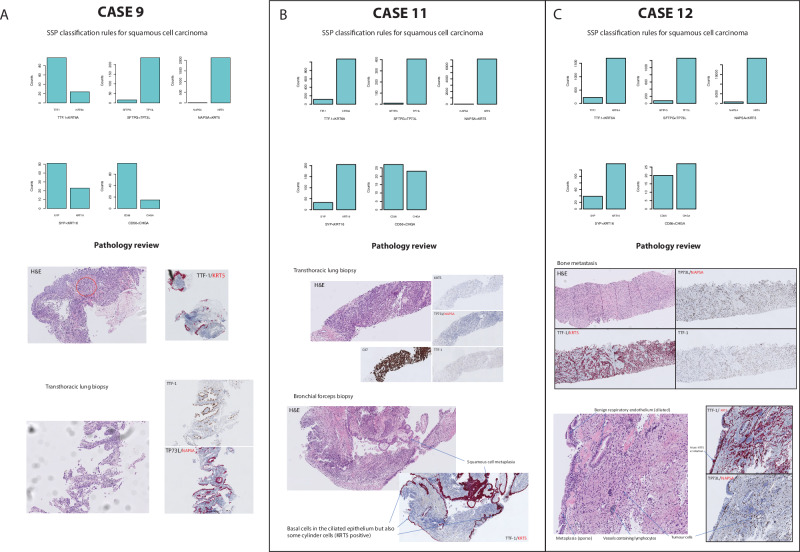
Pathology review of three discrepant cases between NanoString and routine pathology. Histology prediction using a pre-developed SSP and gene expression data derived from NanoString classified three cases (9, 11, and 12) as SqCC. Barplots indicate high expression of genes (*TP73L*, *KRT6A*, *KRT5*, *KRT40*, *KRT16*). Hematoxylin and eosin (H&E) and immunohistochemical (IHC) stains, with DAB (brown) used for single stains, and DAB and Red chromogen (red) for double stains of TTF-1 (brown) in combination with CK5 (red) and p40 (brown) in combination with napsin A (red), respectively. **A** IHC positivity for CK5 is seen in ciliated cells and possible squamous metaplasia of case 9, while the few tumor cells circled in H&E are not seen in the IHC section of the initial biopsy. Follow-up transthoracic biopsy below. **B** Only CK7 was positive in the transthoracic lung biopsy (top) of case 11, while the bronchial biopsy (bottom) showed focal squamous cell metaplasia but no tumor cells. **C** Case 12, with spindle-shaped cells, showed positivity for CK5, p40, and TTF-1, a rare profile that supports handling as adenocarcinoma according to guidelines. SSP single-sample predictor, SqCC squamous cell carcinoma, H&E hematoxylin and eosin, IHC immunohistochemistry, DAB 3,3′-diaminobenzidine, TTF-1 thyroid transcription factor 1, CK5 cytokeratin 5, CK7 cytokeratin 7, p40 ΔNp63, TP73L tumor protein p73-like (p63), KRT keratin (KRT5, KRT6A, KRT16, KRT40).

A histopathological re-review by a thoracic pathologist (H.B.) was performed to analyze the discordance in histology classification between the mRNA-based SSP predictor and the reported clinical histological subtype (Supplementary Table 2). In more than half of discordant cases (*n* = 7/13), the final histological subtype determined in routine clinical practice was based on a specimen from a different tumor lesion obtained later in the diagnostic process, because the initial bronchoscopic specimen was considered insufficient for definitive assessment. Figure 1 illustrates three discrepant cases (discussed in more detail below), for which NanoString analysis proposed an SqCC histology in all cases.

In case 9, the initial bronchoscopic specimen contained sparse malignant cells with a morphology consistent with NSCLC, but the material was insufficient for IHC and molecular profiling. Routine pathology later classified case 9 as AC, based on a follow-up transthoracic core needle biopsy showing clear AC morphology and positive staining for TTF-1 and napsin A. In contrast, the SSP classified the same case as SqCC on the initial bronchoscopy specimen due to high mRNA expression of SqCC-associated genes like *TP73L* and *KRT5*. As shown in Fig. 1A, the tumor cells present in the initial bronchoscopy specimen were scarce (all tumor cells are circled in the H&E stain of Fig. 1A). IHC staining of the initial bronchoscopy specimen revealed positivity for CK5 in ciliated cells and possible focal squamous metaplasia close to the tumor, which may explain the gene expression profile in the bulk RNA used for RNA profiling.

In case 11, the initial bronchoscopy biopsy showed inflammation and focal squamous cell metaplasia but no detectable tumor cells. Basal cells in the ciliated epithelium, as well as some columnar cells in the specimen, were CK5-positive on IHC. The gene expression profile of the initial bronchoscopy specimen was classified as SqCC by the SSP due to high expression of cytokeratin-associated genes (*KRT6A*, *KRT5*, *KRT16*; Fig. 1B). The pathology review of the transthoracic lung biopsy performed later from this patient revealed positivity of only one marker, CK7, while p40, CK5, TTF-1, and napsin A were all negative, and the morphology was non-specific.

The initial bronchoscopy tumor specimen in case 12 had a limited amount of tumor cells positive for CKAE 1/3, TTF-1, and CK5. The diagnosis of NSCLC of unspecific subtype was made due to positivity of the AC-associated marker TTF-1 (clone 8G7G3/1), but also based on IHC positivity of the SqCC-associated markers CK5 and p40 established on a later bone biopsy (Fig. 1C). Interestingly, this IHC profile is reported in less than 1% of all suspected lung cancer cases. Based on spindle-shaped cells, sarcomatoid cancer was suspected, which is a diagnosis that cannot be verified in small specimens. In the clinical setting, it was recommended that this case should be treated as AC based on guidelines for TTF-1 positivity. Based on the high expression of SqCC-associated genes such as *KRT5*, *KRT6A*, and *TP73L*, this case was classified as SqCC by the SSP-classifier.

In contrast to the cases presented above, the pathology review of case 7 confirmed AC with a typical IHC profile in the initial bronchial biopsy. Benign bronchial epithelium was present in the specimen, with no evidence of squamous metaplasia. The SSP analysis indicated high expression of AC-associated genes, including *SFTP*s and *NAPSA*, supporting an AC profile. However, the final class assignment was influenced by stronger rule-based voting for SqCC (*KRT5*, *KRT6A*, and *KRT16*) within the SSP algorithm, as shown in Supplementary Table 1.

### Proteomic analysis of RNAlater-preserved bronchoscopy tissue

**Fig. 2:**
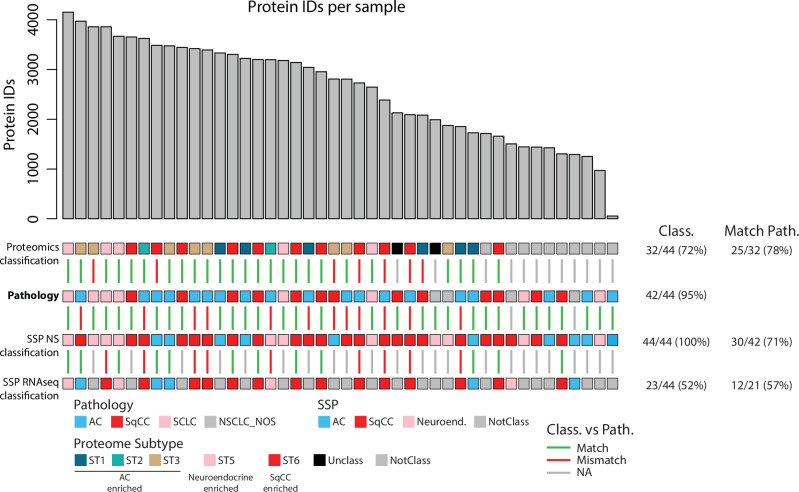
Integrated analysis of gene expression-based classification, proteomics classification, and histology assessment. Comparison between gene expression-based classification using a pre-defined SSP capable of NSCLC histology classification and classification based on proteomics and histology assessment performed in routine clinical diagnostics. Each square represents one unique patient, column-wise, with the histology class represented by color. Each row connecting classified tumors illustrates whether there is a match or mismatch between RNA- or protein-based classification and the pathologist’s histology assessment. SSP single-sample predictor, NS NanoString, RNAseq RNA-sequencing, AC adenocarcinoma, SqCC squamous cell carcinoma, SCLC small cell lung cancer, NSCLC NOS non-small cell lung cancer not otherwise specified, Neuroend. neuroendocrine, NotClass not classified, Unclass unclassified, NA not applicable, ST1–ST6 proteome subtypes (ST1–ST3 adenocarcinoma-enriched, ST5 neuroendocrine-enriched, ST6 squamous cell carcinoma–enriched), Class. classified, Match Path. match with pathology, ID identification.

Included samples analyzed by mass-spectrometry-based proteogenomics in this study are part of a previously reported validation dataset by Lehtiö et al.^17^. In the study by Lehtiö et al., we defined six proteome subtypes in a cohort of 141 early-stage, surgically resected tumors representing all major histological subtypes of NSCLC^17^. For the purpose of the current study, we compared this proteomic classification of tumors to both gene expression-based classification using NanoString-derived data (*n* = 44), RNAseq data (*n* = 23), and routine pathology data. For the comparison, samples proteome-subtyped as ST1, ST2, or ST3 were classified as AC-enriched, ST5 samples as neuroendocrine, and ST6 samples as SqCC, based on the reported characteristics of the proteome subtypes^17^. Twelve tumors could not be classified using proteomics due to the limited number of proteins detected, leaving 32 (73%) overlapping tumors to be compared to NanoString and pathology data, and 20 (45%) to RNAseq data (Table 2, Fig. 2). Concordance and discrepancies in classification versus clinical routine histology assessment are illustrated in Fig. 2 and reported in Table 2. Briefly, the classification based on proteomics had higher concordance with the histology assessment performed in clinical routine than mRNA-based SSP classification. Specifically, 8 of the 12 successfully subtyped ACs that were misclassified by the SSP (including cases 9 and 12 highlighted in Fig. 1) were classified as ACs based on proteomics in concordance with histology assessment in clinical routine.

## Discussion

Precision medicine is a rapidly evolving field that relies on comprehensive molecular profiling of tumors using state-of-the-art technology. Hence, high-quality nucleic acids and proteins will be required to obtain sufficient-quality data and avoid inconclusive test results. Routine diagnostic analyses are often based on FFPE tumor material, which is optimal for IHC-based analyses to assess, for instance, histological subtype and PD-L1 status. However, other treatment-predictive analyses, such as mutation detection and fusion gene detection, using NGS-based techniques, would benefit from nucleic acids of higher quality. This study demonstrates that specimens obtained during lung cancer diagnostics using bronchoscopy with EBUS-TBNA preserved in RNAlater are a source of high-quality nucleic acids. In clinical routine analysis, multiple specimens are often required to confirm the diagnosis and provide the treating physician with information on treatment-predictive mutations, fusion gene status, and histology. Here, we demonstrate the ability to extract RNA, DNA, and proteins from a single bronchoscopy forceps biopsy or EBUS-TBNA specimen preserved in RNAlater to obtain information on gene mutation status, gene fusions, splice variants, histology prediction, and proteomic profiling.

Mutation detection in the current study was compared to results obtained from routine clinical diagnostics. Importantly, for treatment-predictive genes like *EGFR* and *KRAS*, a 100% concordance between the clinical routine NGS analyses and NGS analyses of the RNAlater-preserved LUCAS specimens was observed. Concerning the detection of gene fusions and *MET*ex14 skipping events, two discrepancies were observed. Firstly, one *EML4::ALK* fusion that was detected by clinical routine was not detected by either NanoString or RNAseq analysis of RNA from the LUCAS bronchial forceps tumor biopsy specimen. While the fusion did not reach the pre-set detection threshold in the NanoString assay, manual data inspection did show some support, suggesting a potentially low tumor cell content in the RNAlater-preserved LUCAS biopsy. Clinically, the patient received first-line treatment with a tyrosine kinase inhibitor, but due to poor response, switched to chemotherapy after only four months, and died approximately one year after diagnosis. Secondly, a *MET*ex14 skipping event, undetected by clinical routine on RNA from FFPE samples, was detected using the NanoString assay in a 90-year-old patient who had been deemed unable to receive any antitumor treatment and passed away shortly after diagnosis. Altogether, the analysis of treatment-predictive alterations on the DNA and RNA level suggested good concordance between routine diagnostic procedures performed mainly on FFPE tissue and research-based analysis performed on RNAlater-preserved tissue.

The histological subtypes of lung cancer have been shown to display marked differences in gene expression patterns^11^. Based on this, gene expression-based predictors of lung cancer histology have been proposed, including also predictors capable of predicting single samples (SSPs) using degraded RNA^10,12,13,18^. The conceptual advantage of an SSP is the ability to predict whatever it is trained for in non-normalized single-sample gene expression, irrespective of the platform used for data generation^19^. In the current study, we applied one such SSP of lung cancer histology to RNA extracted from RNAlater-preserved tissue and profiled by both NanoString analysis and RNAseq. The previously trained and validated SSP was developed to distinguish between three distinct histological subtypes of lung cancer: adenocarcinoma, squamous cell carcinoma, and neuroendocrine tumors^18^. Although the sample set of the current study was not limited to the strictly mentioned three categories of lung cancer, but rather encompassed the more representative full spectrum of lung cancer malignancies, the SSP was able to classify samples based on their gene expression profiles and defined SSP classification rules. Notable discordance was observed for both platforms compared to the clinical assessment, with at best an accuracy of 0.61 for the NanoString platform (although balanced accuracies were between 0.74 and 0.75). One possible explanation for the relatively low performance of the SSP predictor could be that it was trained on macro-dissected FFPE material rather than RNAlater-preserved bulk specimens that may contain more infiltrating non-malignant cell types. Of importance, the algorithm used for the pre-trained and validated SSP uses equal amounts of gene rules for multi-class classification, irrespective of the importance of the selected genes for the three histological subtypes (Supplementary Table 1). To exemplify, neuroendocrine lung cancer classification includes expression of *KRT6A* and *KRT40*, two genes strongly associated with a squamous cell lung cancer histology, yet these still influence the classification of neuroendocrine lung cancer using the current SSP^18,19^. Interestingly, comparison of protein-based histology classification using a two-class algorithm (kTSP) with pathologists’ subtyping also failed to achieve complete concordance but performed slightly better than the mRNA-based SSP. Importantly, proteomics, NanoString, and RNAseq measure protein or gene expression from bulk tumor tissue. As a result, the generated profiles reflect a mixture of signals from different cell types present in the tumor and their relative proportions. In contrast, analyses based on FFPE tissue sections and IHC provide spatial and morphological information, allowing cell type-specific interpretation. For example, squamous metaplasia within an adenocarcinoma can lead to increased expression of SqCC-associated genes, such as KRT5 and TP73L, in NanoString and proteomics data, as observed when discrepant cases in this study were re-reviewed. Therefore, for bulk tissue-based methods to be clinically informative, adequate tissue representativity is critical. A limitation of the present study is that routine and research-based histological assessments were not always performed on the same tissue specimens (Supplementary Table 3). In some cases, histological subtyping in clinical practice was based on tissue obtained from subsequent diagnostic procedures because material from the initial bronchoscopy was insufficient for definitive diagnosis. It could therefore be argued that greater concordance between our SSP and the routine clinical assessment of histology could have been achieved if we had repeated our analysis on the material that later turned out to be the diagnostic one. Importantly, clinical guidelines for assessing histology in routine diagnostics could also be a potential explanation for some discrepancies, e.g., AC diagnosis based on the positivity of a single AC marker in tumors where the SqCC components highly affect the SSP classification. Altogether, the performance of the predictor suggests that histological subtyping using bulk RNA and gene expression could serve as a valuable complementary tool to standard clinical histopathological techniques, given further validation.

Unlike the FFPE workflow, where sections for IHC and morphology differ from sections used for nucleic acids extraction, the research approach presented in this study ensures that nucleic acids and proteins are all extracted from the same tumor specimen. Notably, proteins extracted from the specimens included in this study proved to be of good quality and appropriate for MS-based proteomic analysis.

Proteins extracted from bulk tissue dilute tumor-specific proteins, which could explain the substantial proportion of samples (12/44) left unclassified. However, albumin detection (Supplementary Fig. 1) indicates high variability in protein content, with low inter-sample variation in protein detection. Nevertheless, the successful acquisition and use of proteins in the current study provide an opportunity to move proteomic-based methods potentially closer to clinical use, without compromising DNA or RNA quality or requiring additional tissue sampling.

In conclusion, RNAlater-preserved bronchoscopy and EBUS-TBNA specimens obtained during initial lung cancer diagnostics provide high-quality nucleic acids and proteins suitable for advanced multi-omics analyses, including genomics, transcriptomics, and mass-spectrometry-based proteomics. This approach provides a basis for comprehensive multi-omics investigations from a single tumor specimen, supporting the development of more advanced diagnostic assays in lung cancer despite limited tissue availability.

## Methods

### Ethics statement

The Regional Ethical Review Board at Lund University approved the Lung Cancer Study in Southern Sweden (LUCAS) study in adherence to the Declaration of Helsinki^20^. (Registration no. 2014/32). All patients provided written informed consent prior to enrollment. The study followed patient consent and ethical review board regulations.

### Patients

In the LUCAS study, blood, cytology specimens, and tumor tissue from patients with suspected or confirmed lung cancer are biobanked for research purposes. These samples are collected during routine diagnostic procedures, such as bronchoscopy, surgical interventions, and longitudinal blood sampling during lung cancer treatment and follow-up.

Between January 18, 2019, and January 31, 2020, 75 LUCAS patients at the Unit for Interventional Pulmonology (IP) at the Skåne University Hospital in Lund, Sweden, were sampled for research purposes during diagnostic bronchoscopy with EBUS-TBNA for suspected lung cancer. All patients had a confirmed pulmonary mass and/or mediastinal lymphadenopathy when previously examined with computed tomography of the chest (chest CT), supplemented by a positron emission tomography-computed tomography (PET-CT) when indicated. Review of medical and pathology records of the 75 patients identified three benign cases, seven metastatic non-primary lung cancer cases, and one case with inadequate cytology that was lost to follow-up. This left 64 patients diagnosed with primary lung cancer. Based on routine pathology analysis, 26 of 64 cases were classified as adenocarcinoma (AC), including one patient later lost to follow-up, 17 as squamous cell carcinoma (SqCC), 10 as small cell lung carcinomas (SCLC), while 11 cases were classified as non-small cell lung cancer not otherwise specified (NSCLC NOS). After excluding patients with benign findings (*n* = 3), non-primary lung cancer (*n* = 7), patients lost to follow-up (*n* = 2), and patients with operable lung cancer (*n* = 10), a final cohort of 53 patients with non-resectable lung cancer was defined for further studies.

### Analytical workflow

**Fig. 3:**
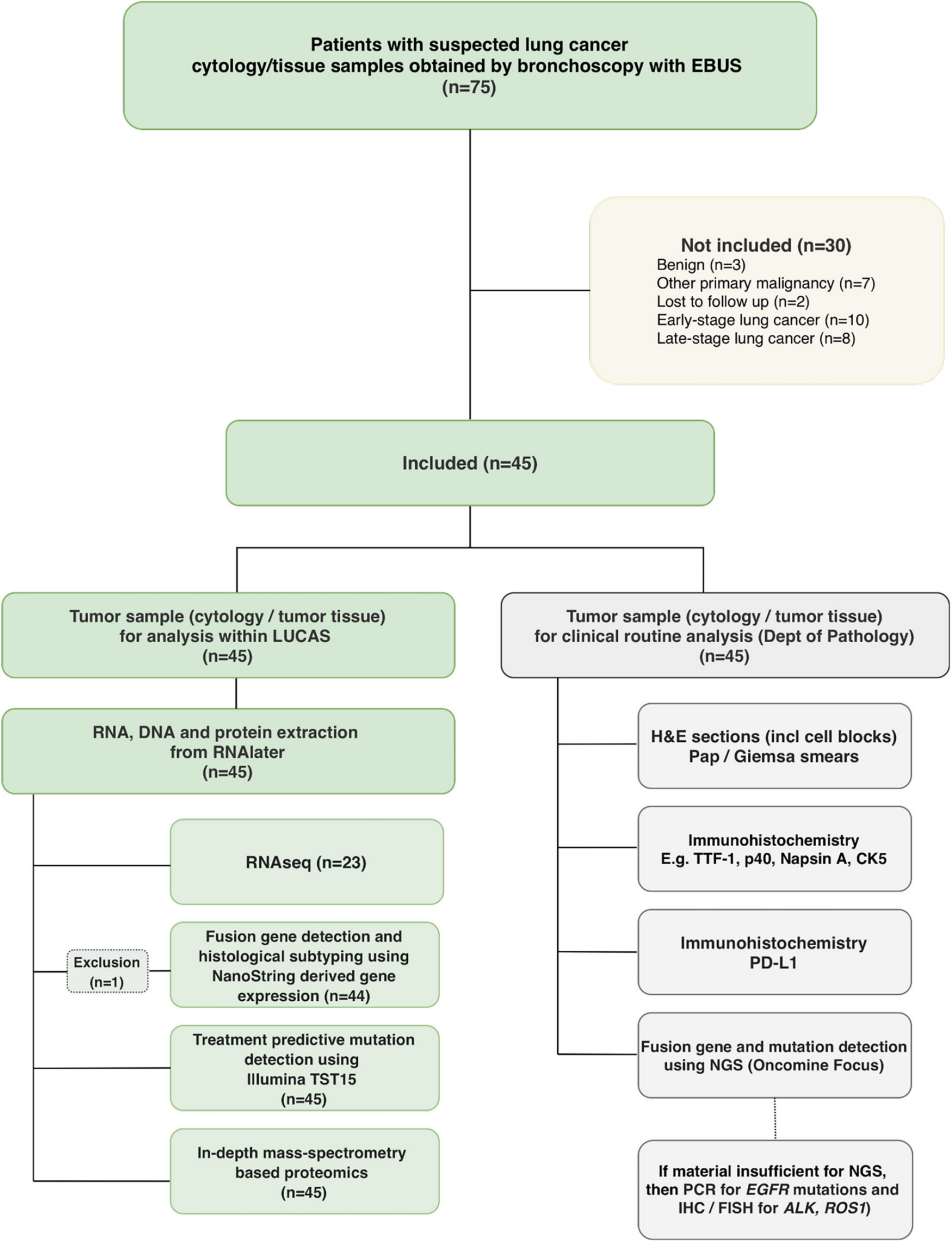
Overview of patient inclusion and analysis performed in the study. From January 18th, 2019, to January 31st, 2020, 75 patients enrolled in the LUCAS study underwent bronchoscopy with EBUS-TBNA at the Unit for Interventional Pulmonology at the Skåne University Hospital for suspected lung cancer. From all non-resectable lung cancer cases (53 in total), we selected 45 to ensure representation of all histological types for gene expression analysis, mutation screening, and proteomic profiling. EBUS, endobronchial ultrasound (–guided transbronchial needle aspiration, EBUS-TBNA), LUCAS Lung Cancer Study in Southern Sweden, RNAseq RNA-sequencing, TST-15 TruSight Tumor 15 (Illumina) panel, H&E hematoxylin and eosin, Pap Papanicolaou, TTF-1 thyroid transcription factor 1, CK5 cytokeratin 5, PD-L1 programmed death-ligand 1, NGS next-generation sequencing, PCR polymerase chain reaction, IHC immunohistochemistry, FISH fluorescence in situ hybridization, EGFR epidermal growth factor receptor, ALK anaplastic lymphoma kinase, ROS1 ROS proto-oncogene 1.

From the 53-patient cohort, 45 cases representing all lung cancer histological types (AC, SqCC, SCLC, and NSCLC NOS) were selected for final analyses by our research laboratory (Division of Oncology, Department of Clinical Sciences, Lund University, Sweden). All research-derived results were compared to corresponding results from clinical routine analyses performed at the time of diagnosis (Department of Pathology, Skåne University Hospital, Lund, Sweden). A flowchart of patient inclusion and performed analyses is shown in Fig. 3. Table 2 presents the baseline characteristics of the cohort. The final patient database includes patient demographic information, clinical characteristics, TNM status determined by CT and PET-CT scans, tumor location, sampled lymph nodes, tumor specimens obtained from bronchoscopy with EBUS-TBNA, histopathological diagnosis, mutation and fusion gene status, and treatment follow-up data.

**Table 2:** Baseline characteristics of the cohort and specification of sample types

Baseline patients’ characteristics		Number	Percent
**Total number of patients**		45	100%
**Mean age**		74.4	
**Sex**			
	Female	23	51%
	Male	22	49%
**Smoking**			
	Current smoking	14	31%
	Former smokers	28	62%
	Never-smokers	3	7%
**Site of primary tumor localization**			
	Right Upper Lobe (RUL)	12	27%
	Right Middle Lobe (RML)	3	7%
	Right Lower Lobe (RLL)	5	11%
	Left Upper Lobe (LUL)	13	29%
	Left Lower Lobe (LLL)	12	27%
**cTNM**			
	Stage I	0	0%
	Stage II	2	4%
	Stage III	14	31%
	Stage IV	29	64%
**Analyzed specimen type**		**Clinical routine**	**Study**
**Total number of patients**		45	45
	EBUS-TBNA cytology only	6	9
	Forceps biopsy only	20	36
	EBUS-TBNA cytology + forceps biopsy	19	0

### Sampling

Cytology and tumor tissue were collected using bronchoscopy and EBUS-TBNA technique as part of the clinical routine at the IP unit at the Skåne University Hospital in Lund, Sweden, as described by Herth et al.^4^. All bronchoscopic procedures were performed on an outpatient basis, utilizing topical anesthesia and monitored moderate conscious sedation. The location of the pulmonary lesion dictated whether a conventional or advanced bronchoscopy was used. For patients with mediastinal/hilar lymphadenopathy or centrally located pulmonary lesions, EBUS-TBNA was subsequently performed. The choice of bronchoscope size and model was determined by the operator based on lesion characteristics (Olympus BF-1TH190; BF-P190; BF-MP190; Olympus Corp., Tokyo, Japan). For EBUS-TBNA, a convex probe ultrasound flexible video bronchoscope was used (Olympus BF-UC 180F; Olympus Corp., Tokyo, Japan). Tissue samples were obtained with biopsy forceps (EndoJaw, Olympus FB-211D.A/221D.A/231D.A/241D.A), and fine-needle aspiration with 22-gauge or 21-gauge EBUS-TBNA needles (Olympus ViziShot, NA-U401SX-4022, and NA-201SX-4022). All procedures in the current study were performed by four experienced operators (M.K.-K., S.B., J.K., A.N). Every procedure began with a conventional bronchoscopy to examine the lower respiratory tract. Tumor masses visible in the central airways were sampled with bronchial brushes and forceps. For peripheral pulmonary lesions, advanced techniques, such as thin and ultrathin bronchoscopes, radial EBUS, fluoroscopy, and electromagnetic navigational bronchoscopy (ENB), were applied. When indicated, EBUS-TBNA bronchoscopy was performed during the same session, immediately after the diagnostic bronchoscopy. Mediastinal lesions, pathologically enlarged lymph nodes, and FDG-avid lymph nodes were visualized and sampled in real-time using EBUS-TBNA, and the specimens were prepared for analysis according to recommendations in the guideline for the acquisition and preparation of EBUS-TBNA specimens for diagnostic and molecular testing^5^. In all EBUS-TBNA procedures, rapid on-site evaluation (ROSE) was used to verify sample adequacy by assessing the presence of sufficient lymphocytes in the smears. During each bronchoscopy, tumor material needed for the clinical routine diagnostics was obtained first and placed in CytoLyt® and/or formalin, respectively, and handled according to established protocols for clinical diagnostics. Thereafter, additional tumor specimens (bronchoscopic forceps biopsies and EBUS-TBNA cytology specimens) were collected from the same anatomical tumor location for research purposes. These research specimens were preserved in RNAlater (cat no AM7020, Invitrogen, Waltham, MA, USA) and deposited in the LUCAS Biobank (Fig. 3).

### Nucleic acids and protein extraction

RNA, DNA, and proteins were extracted from RNAlater-preserved tissue using the AllPrep DNA/RNA Mini Kit (catalog number 80204, Qiagen, Hilden, Germany). RNA was quantified using the NanoDrop (ThermoFisher Scientific, Waltham, MA, USA) and evaluated on the Bioanalyzer (Agilent Technologies, Santa Clara, CA, USA). DNA was quantified using the Qubit Fluorometric Quantification system (ThermoFisher Scientific). Protein-containing flowthrough was collected during extraction. For clinical routine diagnostic analyses, DNA and RNA were extracted according to standard protocols.

### NanoString gene expression analysis

NanoString analysis was performed using 100 ng RNA as input from the 45 selected RNAlater-preserved specimens using an RNA-based nCounter Elements assay (NanoString Technologies, Seattle, WA, USA). A previously designed probe set was used on a Sprint instrument according to the manufacturer’s instructions^18^. Fusion-positive controls were included, and fusion gene prediction was performed analytically as previously described^21,22^. Quality control using the NanoString provided nSolver software and thresholds set by the manufacturer deemed one sample (*n* = 1) to be of insufficient quality. This sample was excluded, and 44 samples were included for further analysis. Fusion genes (involving the genes *ALK*, *RET*, *ROS1*, *NRG1,* and *NTRK1*) and *MET*ex14 skipping events were detected as previously described^18,21,22^. During the study period, 2019–2020, gene fusion detection in routine clinical practice was primarily performed using the Oncomine™ Focus Assay on the Ion Torrent S5™ platform as the first-line RNA-based method. If this approach was unsuccessful, IHC for *ALK* and *ROS1* was used as a second-line method for biopsy specimens, with fluorescence in situ hybridization (FISH) as a third-line option. For cytology specimens, FISH was used as the second-line method, as *ALK* and *ROS1* IHC were not validated for cytology preparations during the inclusion period.

### RNA-sequencing

Gene expression data from 23 patients included in the present study, previously selected based on histological subtyping to represent the subtypes adenocarcinoma, squamous cell carcinoma, and neuroendocrine tumors, were available through another study^23^. To identify potential gene fusions, the FusionCatcher v1.33 tool was used, with default settings^24^ and the GRCh38/hg38 build as a human reference genome.

### Mutation detection

20 ng DNA from research specimens was used as input to the next-generation sequencing (NGS)-based TST-15 mutation screening panel (Illumina Inc.). Library preparation was performed according to the manufacturer’s instructions and sequenced on a MiSeq instrument (Illumina Inc.). Generated VCF files were imported to VariantStudio for variant calling. Mutation analysis in clinical routine during the study period was predominantly performed using the Oncomine Focus Assay (ThermoFisher Scientific, Waltham, MA, USA).

### Prediction of NSCLC histology from gene expression data

A single-sample predictor (SSP) previously developed and trained to identify NSCLC histology was applied to gene expression data derived from NanoString and RNA-sequencing (RNAseq)^18^. The SSP was trained on NanoString-derived gene expression data from FFPE-extracted RNA and tested in independent gene expression cohorts generated by different gene expression platforms and a variety of RNA sources, demonstrating high accuracy. Decision-based gene rules for classification are stated in Supplementary Table 1. Briefly, each gene-pair rule listed above is evaluated within the sample. Each rule provides evidence for a particular subtype. The results are combined using a Naive Bayes classifier, and the subtype with the highest posterior probability is assigned a classification label. The SSP was applied to background-corrected, non-normalized gene expression data generated by NanoString (*n* = 44) and RNAseq (*n* = 23). SSP prediction was compared with routine diagnostic histology assessment performed in the Pathology department at Skåne University Hospital during the study period. Discrepant cases were reviewed by a thoracic pathologist (H.B.) using hematoxylin and eosin (H&E) and immunohistochemical (IHC) stains according to standard procedures.

### Proteomics analysis

The protein-containing flowthrough from all 44 patients has previously been used to study the proteome using in-depth mass-spectrometry-based (MS) proteomic analysis^17^. The obtained quantitative proteomics data covered 4154 proteins in total, with a median of 2808 quantified proteins per sample^17^. The number of identified proteins per sample showed a negative correlation to serum albumin levels as quantified in the MS-data, indicating that blood or plasma protein levels remaining in the sample affect the quality of the MS-data (Supplementary Fig. 1). Samples were classified based on MS-data into six different proteome subtypes (ST1, ST2, ST3, ST4, ST5, and ST6) using a k-Top Scoring Pairs (kTSP)^25^ classifier described by Lehtiö et al.^17^. Due to a low number of identified proteins and a lack of coverage of kTSP features required for classification, 10 samples were filtered out before classification (“Not classified”). For two samples, the kTSP classifier did not output a clear classification (“Unclassified”), leaving, in the end, 32 samples with a proteomic subtype class.

## Supplementary information

Supplementary Information

## Data Availability

Several data items used in the analyses performed in this article are available from original publications. The previously reported RNAlater RNA-sequencing data of stage IV tumors from Staaf et al.^23^ used in this study is available as raw FPKM data from the deposited supplementary data in the original study. The previously reported proteomic data from Lethiö et al.^17^ used in this study is available from the repositories listed in the original study. All detected and filtered somatic variants from the Illumina TST-15 analysis for relevant samples are reported in Supplementary Table 4. Raw VCF files are available from the corresponding author on reasonable request due to size constraints. Raw Nanostring count data used in this study is available in Supplementary Table 5.

## Code availability

No specific code was developed for this project.
