# Supplementary material for: Integrated prediction of lung cancer histology and molecular profiles from small bronchoscopic tumor specimens
Source: NPJ Precis Oncol. 2026 Jul 27;10:292. doi: 10.1038/s41698-026-01623-7 (PMC13408671; doi:10.1038/s41698-026-01623-7)
Supplement: Supplementary file 1 — Supplementary Information [file 41698_2026_1623_MOESM1_ESM.pdf]

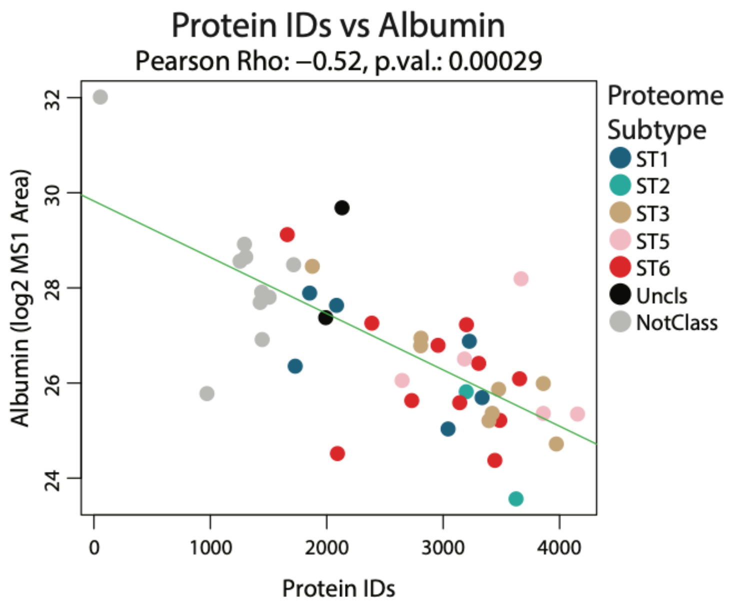

Supplementary figure 1. Protein IDs vs Albumin. Detected proteins using in-depth mass spectrometry was correlated to albumin detection for all 33 included samples.

GENE EXPRESSION*	CLASSIFICATION LABEL
KRT5<SFTPG	Adenocarcinoma
KRT6A<TTF-1	Adenocarcinoma
NAPSA<TP73L	Adenocarcinoma
CD56<CHGA	Adenocarcinoma
KRT16<SYP	Adenocarcinoma
SFTPG<TP73L	Squamous cell carcinoma
NAPSA<KRT5	Squamous cell carcinoma
TTF-1<KRT6A	Squamous cell carcinoma
SYP<KRT16	Squamous cell carcinoma
CD56<CHGA	Squamous cell carcinoma
CD56<TP73L	Neuroendocrine carcinoma
CHGA<KRT5	Neuroendocrine carcinoma
SFTPG<TTF-1	Neuroendocrine carcinoma
NAPSA<SYP	Neuroendocrine carcinoma
KRT40<KRT6A	Neuroendocrine carcinoma

*Expression of gene 1 lower (<) than gene 2

Supplementary Table 1. Single sample predictor classification rules
Non-normalized gene expression data is used for SSP classification. Each gene-pair rules listed above are evaluated within the sample. Each rule provides evidence for a particular subtype (classification label).The results are combined using a Naive Bayes classifier and the subtype with the highest posterior probability is assigned a classification label. Abbreviations: SSP, single sample predictor; TTF-1, thyroid transcription factor 1; KRT5, cytokeratin 5; SFTPG, Surfactant Associated Protein G; KRT6A, cytokeratin 6A; NAPSA, Napsin A, TP73L, Tumor Protein P63; CD56, Neural Cell Adhesion Molecule 1; CHGA, Chromogranin A.

			Gene expression of adenocarcinoma related markers			Gene expression of squamous cell carcinoma related markers					Gene expression of neuroendocrine related markers			SSP classification. Total number of rules for each class N=5							
**Case**	**Specimen for patology review**	**Specimen for SSP**	*NAPSA*	*SFTPG*	*TTF-1*	*KRT40*	*KRT6A*	*TP73L*	*KRT5*	*KRT16*	*SYP*	*CHGA*	*CD56*	**No of adenocarcinoma class rules fulfilled**	**No of squamous cell carcinoma class rules fulfilled**	**No of neuroendocrine class rules fulfilled**	**SSP histology predicition outcome** (bronchoscopy specimen)	**Pathology department outcome** (bronchoscopy specimen)	**Pathology department final diagnosis**	**Pathology department review**	**PATHOLOGY department review**
1	BB RUL	BB RUL	High	High	Low	Low	Low	Low	Low	Low	Low	Low	Low	5	0	0	Adenocarcinoma	Benign bronchial biopsy	Squamous cell carcinoma (dg later on bronchoscopic rebiopsy)	Squamous cell carcinoma	BB essentially benign with a lot of macrophages and some type II pneumocytes (Napsin A and TTF-1positive)
2	BB LUL	BB LUL	Low	Low	High	Low	Low	High	High	Low	Low	Low	Low	3	2	3	Neuroendocrine	Benign bronchial biopsy	Adenocarcinoma (on cytology sample from bronchial brush)	NSCLC NOS	BB with benign tissue (some inflammation) NSCLC in cytology (bronchial brush) but insufficient for further analysis (it was suggested that "adenocarcinoma cannot be ruled out")
3	BB LUL	BB LUL	High	Low	Low	Low	High	Low	Low	Low	Low	Low	Low	2	3	3	Squamous cell carcinoma	Adenocarcinoma	Adenocarcinoma	Adenocarcinoma	BB with adenocarcinoma with typical IHC profile, quite a lot of tumor, practically no bronchial epithelium (no metaplasia)
4	BB LUL	BB LUL	Low	High	Low	Low	High	Low	Low	High	Low	Low	Low	1	3	0	Squamous cell carcinoma	Adenocarcinoma	Adenocarcinoma	Adenocarcinoma	BB with adenocarcinoma with typical IHC profile, moderate amount of tumor, prctically no bronchial epithelium (no metaplasia), inflammation is present
5	SB RUL	BB RUL	Low	Low	High	Low	Low	High	High	Low	High	Low	Low	3	3	2	Squamous cell carcinoma	Benign bronchial biopsy	Adenocarcinoma (dg later on surgical lung biopsy)	Adenocarcinoma	Initial BB without malignancy but a lot of epithelium (no metaplasia). SB later with adenocarcinoma (with signet ring morphology, only CK7 pos, p40 neg, CK5 not stained)
6	FNA EBUS LN 7	FNA lymph node 7	High	High	High	Low	Low	Low	High	High	Low	Low	Low	2	3	1	Squamous cell carcinoma	Lymph node FNA cytology insufficient for diagnosis	Adenocarcinoma (dg later on skin met)	Adenocarcinoma	FNA mostly necrosis and some atypical cells (insufficient for diagnosis). Skin excision metastasis of adenocarcinoma positive for TTF-1
7	BB RLL	BB RLL	High	High	Low	Low	High	Low	High	High	Low	Low	Low	1	3	0	Squamous cell carcinoma	Adenocarcinoma	Adenocarcinoma	Adenocarcinoma	BB with adenocarcinoma with typical IHC profile, quite a lot of tumor, there is benign bronchial epithelium (no metaplasia)
8	BB LLL	BB LLL	Low	Low	Low	Low	High	High	High	High	Low	Low	Low	1	4	0	Squamous cell carcinoma	Adenocarcinoma	Adenocarcinoma	Adenocarcinoma	BB with adenocarcinoma with typical IHC profile, a lot of tumor, practically no bronchial epithelium (no metaplasia)
9	BB RUL	BB RUL	Low	Low	High	Low	Low	High	High	Low	High	Low	Low	3	3	2	Squamous cell carcinoma	Bronchial biopsy insufficient for definite diagnosis	Adenocarcinoma (dg later on TTP lung biopsy)	Adenocarcinoma	BB with scant tumor cells suggests NSCLC morphology without definite metaplasia, insufficient for IHC. TTP biopsy later confirms adenocarcinoma with typical IHC profile
10	FNA EBUS LN 4R	FNA lymph node 4R	Low	Low	High	Low	Low	High	High	Low	High	Low	Low	3	3	2	Squamous cell carcinoma	Adenocarcinoma	Adenocarcinoma	Adenocarcinoma	FNA with adenocarcinoma with typical IHC profile (EGFR L858R)
11	BB LUL	BB LUL	Low	Low	Low	Low	High	High	High	High	Low	Low	Low	1	4	2	Squamous cell carcinoma	Benign bronchial biopsy with cell metaplasia	Adenocarcinoma (dg later on TTP lung biopsy)	NSCLC NOS	BB without malignancy, metaplasia is seen. TTP later with NSCLC only CK7+ (KRAS G12V)
12	BB RLL	BB RLL	Low	Low	Low	Low	High	High	High	High	Low	Low	Low	0	5	1	Squamous cell carcinoma	Malignancy	Adenocarcinoma (dg later on bone met)	NSCLC of unclear type	BB with NSCLC of unclear type, CK5, p40, and TTF-1 in all cells, spindle shaped; handling as adenocarcinoma recommended, but not definite subtyping
13	BB RLL	BB RLL	High	Low	High	Low	Low	High	Low	High	Low	Low	Low	3	3	1	Squamous cell carcinoma	Adenocarcinoma	Adenocarcinoma	Adenocarcinoma	Adenocarcinoma with typical IHC profile (and known EGFR delex19), CK5 not stained as not deemed necessary, one biopsy with tumor, one benign with normal epithelium (no metaplasia)

Supplementary table 2. Review of the cases where the pathology department and SSP predictor outcomes differ. Abbreviations: BB, Bronchial (forceps) biopsy provided by bronchoscopy; SB, Surgical biopsy; FNA, Fine needle aspiration by EBUS; TTP, Transthoracic lung biopsy; LN, Lymph node; RUL, Right upper lobe; RLL, Right lower lobe; LUL, Left upper lobe; NSCLC NOS, Non-small cell lung cancer not otherwise

ID	Sampling		Clinical management		
	Sampling type	Location	cTNM	ECOG	Treatment
1	Forceps biopsy	Left lower lobe	T2aN3M0	2	Chemotherapy + radiotherapy
2	Forceps biopsy	Left upper lobe	T4N3M1b	2	Chemotherapy
3	Forceps biopsy	Left upper lobe	T4NxM1b	2	Chemotherapy + radiotherapy
4	Forceps biopsy	Right upper lobe	T4N3M1c	3	No
5	Forceps biopsy	Left upper lobe	T4N0M0	2	Chemotherapy + immunotherapy
6	Forceps biopsy	Left lower lobe	T4N2M1b	1	Chemotherapy
7	Forceps biopsy	Left upper lobe	T3N2M0	1	Chemotherapy + radiotherapy
8	Forceps biopsy	Left lower lobe	T3N1M1b	1	Chemotherapy + immunotherapy
9	FNA	Right upper lobe	T4N2M1b	1	No
10	Forceps biopsy	Left lower lobe	T4N2M0	1	Chemotherapy + radiotherapy
11	Forceps biopsy	Right upper lobe	T2aN2M0	1	Chemotherapy + radiotherapy
12	Forceps biopsy	Left upper lobe	T3NxM0	1	Chemotherapy + radiotherapy
13	Forceps biopsy	Left upper lobe	T3N3M1b	X	No
14	Forceps biopsy	Left lower lobe	T1cN1M0	1	Chemotherapy + radiotherapy
15	Forceps biopsy	Right upper lobe	T4N2M1c	1	Chemotherapy + radiotherapy
16	Forceps biopsy	Left upper lobe	T1cN3M0	0	Chemotherapy + radiotherapy
17	Forceps biopsy	Left upper lobe	T4N0M1b	0	Chemotherapy + radiotherapy
18	FNA	EBUS pos 7	T4N2M1c	3	No
19	Forceps biopsy	Right lower lobe	T2aN2M1b	0	Chemotherapy + immunotherapy
20	Forceps biopsy	Left lower lobe	T1cN2M0	1	Chemotherapy + radiotherapy
21	FNA	EBUS pos 7	T1cN2M1c	4	Radiotherapy
22	Forceps biopsy	Left upper lobe	T1cN2M0	2	Chemotherapy
23	Forceps biopsy	Left upper lobe	T2bN0M0	1	Chemotherapy + radiotherapy
24	FNA	EBUS pos 11R	T4N2bM1c	1	Chemotherapy
25	Forceps biopsy	Right middle lobe	T4N2M1b	1	Chemotherapy
26	Forceps biopsy	Right upper lobe	T4N0M0	2	Immunotherapy
27	Forceps biopsy	Left lower lobe	T2bN1M1c	1	Chemotherapy + radiotherapy
28	FNA	EBUS pos 7	T4N3M1c	0	Chemotherapy + radiotherapy
29	Forceps biopsy	Right lower lobe	T4N3M1c	2	Tyrosine kinase inhibitor
30	Forceps biopsy	Right upper lobe	T2bN2M1b	1	Chemotherapy
31	Forceps biopsy	Left lower lobe	T4N1M0	0	Chemotherapy + radiotherapy + surgery
32	Forceps biopsy	Left lower lobe	T4N3M1a	3	No
33	Forceps biopsy	Left upper lobe	T2N3M1c	0	Tyrosine kinase inhibitor
34	Forceps biopsy	Left lower lobe	T3N3NM1c	0	Immunotherapy
35	Forceps biopsy	Right upper lobe	T1bN2M0	0	Chemotherapy + radiotherapy
36	FNA	EBUS pos 4R	T4N2M0	3	Chemotherapy + radiotherapy
37	Forceps biopsy	Right upper lobe	T2aNxM1b	1	No
38	Forceps biopsy	Left lower lobe	T2aN2M1a	2	Immunotherapy
39	FNA	EBUS pos 4R	T4N2M0	1	Tyrosine kinase inhibitor
40	FNA	EBUS pos 10R	T4N1M1c	3	No
41	Forceps biopsy	Left upper lobe	T4N2M1c	1	Immunotherapy
42	Forceps biopsy	Right lower lobe	T4N2M1c	3	No
43	FNA	EBUS pos 7 and 4 R	T1bN2MO	1	Chemotherapy
44	Forceps biopsy	Right lower lobe	T2aN3M1b	2	Tyrosine kinase inhibitor
45	Forceps biopsy	Left upper lobe	T1bN2M1b	3	Chemotherapy + radiotherapy

ID	Histopathological and treatment predictive analysis — Immunhistochemical stainings performed on diagnostic specimen (FFPE)													
	CKAE1/3	CK7	TTF1	NAPSA	KRT40	PAX8	CK5	TP63	SYP	CD56	KI67	PDL1	Initial histological subtype	Tumor cell content
1	Positive		Positive						Positive		High	NA	SCLC	NA
2			Negative				Positive					Negative	SqCC	NA
3			Positive	Positive	Negative							Negative	AC	NA
4		Positive	Negative	Negative	Negative	Negative	Negative					Negative	NOS	NA
5				Negative	Positive							25-49%	SqCC	50%
6			Positive	Positive	Negative				Negative	Negative		1-4%	AC	NA
7	Positive		Positive	Negative	Negative		Negative		Positive	Positive		1-4%	SCLC	NA
8		Positive	Negative	Negative	Positive		Positive	Positive				1-4%	SqCC	NA
9												50-74%	NOS	NA
10												Negative	AC	60%
11			Negative	Negative	Positive		Positive					Inconclusive	SqCC	NA
12												NA	AC	NA
13			Positive	Positive	Positive							50-74%	AC	NA
14	Positive		Positive						Positive	Positive		NA	SCLC	NA
15	Positive		Positive		Negative				Positive	Positive	High	NA	SCLC	NA
16			Positive	Positive	Negative		Negative					Negative	AC	NA
17												1-4%	AC	NA
18	Positive		Positive						Positive	Positive		NA	SCLC	NA
19		Positive	Negative	Negative	Positive							50-74%	SqCC	NA
20	Positive		Positive						Positive	Positive		NA	SCLC	NA
21		Positive	Negative	Negative	Negative							Negative	AC	NA
22												NA	SqCC	NA
23												NA	SqCC	NA
24												1-4%	AC	50%
25			Negative	Negative	Positive		Positive					1-4%	SqCC	NA
26												>75%	AC	NA
27	Positive										High	NA	SCLC	NA
28	Positive	Positive	Negative	Negative	Negative	Negative	Inconclusive	Inconclusive				Negative	AC	NA
29												NA	AC	60%
30												1-4%	SqCC	30%
31	0	0	Positive	Positive	Negative	0	Negative	0				1-4%	SqCC	40%
32												1-4%	AC	NA
33	0	0	Positive	Positive	Negative	0	Negative	0				NA	AC	40%
34	0	Positive	Positive	Positive	Negative	0	Negative	0				50-74%	AC	25%
35												Negative	SqCC	15%
36	0	0	Negative	Negative	Positive	0	Positive	0				>50%	SqCC	15%
37												Negative	AC	20%
38	0	0	Negative	Negative	Positive	0	Positive	0				<75%	SqCC	15%
39												NA	AC	40%
40	0	0	Positive	0	Negative	0	Negative	0	Positive	Positive	High	NA	SCLC	80%
41												>75%	AC	90%
42	Positive	0	Positive	Inconclusive	Inconclusive	0	Positive	0				Negative	AC	NA
43	0	Positive	Positive	0	Negative	0	Negative	0				>75%	AC	NA
44												NA	AC	40%
45	0	0	Positive	Positive	Negative	0	0	0				25-49%	AC	30%

ID	Histopathological and treatment predictive analysis — Analysis performed on diagnostic specimen (FFPE)						
	Oncogenetics using Oncomine Focus NGS panel	*ALK* fusion	*ROS1* fusion	*RET* fusion	*MET* ex14 skipping event	*EGFR* mutation	*KRAS* mutation
1	Negative	No	No	No	No	No	No
2	Negative	No	No	No	No	No	No
3	Negative	No	No	No	No	No	Yes
4	Negative	No	No	No	No	No	No
5	Negative	No	No	No	No	No	No
6	Negative	No	No	No	No	No	No
7	Negative	No	No	No	No	No	No
8	Negative	No	No	No	No	No	No
9	Negative	No	No	No	No	No	No
10	Positive	No	No	No	No	Yes	No
11	Negative	No	No	No	No	No	No
12	NA	NA	NA	No	No	NA	NA
13	Negative	No	No	No	No	No	Yes
14	NA	No	No	No	No	No	No
15	NA	NA	NA	No	No	NA	NA
16	NA	NA	NA	No	No	NA	NA
17	Negative	No	No	No	No	Yes	No
18	NA	NA	NA	No	No	NA	NA
19	Negative	No	No	No	No	No	No
20	NA	NA	NA	No	No	NA	NA
21	Negative	No	No	No	No	No	No
22	Inconclusive	Inconclusive	Inconclusive	No	No	No	No
23	NA	No	No	No	No	No	No
24	Negative	No	No	No	No	No	Yes
25	Negative	No	No	No	No	No	Yes
26	Negative	No	No	No	No	No	No
27	Not done	No	No	No	No	No	No
28	Negative	No	No	No	No	No	No
29	Positive	Pos	No	No	No	No	No
30	Negative	No	No	No	No	No	No
31	Negative	No	No	No	No	No	No
32	Negative	No	No	No	No	No	No
33	Positive	No	No	No	No	Yes	No
34	Negative	No	No	No	No	No	No
35	Negative	No	No	No	No	No	No
36	Negative	No	No	No	No	No	No
37	Inconclusive	No	Inconclusive	No	No	No	No
38	Negative	No	No	No	No	No	No
39	Positive	No	No	No	No	Yes	No
40	Negative	No	No	No	No	No	No
41	Negative	No	No	No	No	No	No
42	Negative	No	No	No	No	No	No
43	Negative	No	No	No	No	No	No
44	Positive	No	No	No	No	Yes	No
45	Negative	No	No	No	No	No	No

ID	Study specific analysis on RNAlater preserved small bronchoscopic tumor specimens — Gene expression analysis		Results from gene expression analysis using NanoString					Mass-spectrometry based proteomics		Massive parallell sequencing based mutation detection using Illumina TST-15		
	NanoString	RNA sequencing	*ALK* fusion	*ROS1* fusion	*RET* fusion	*MET* ex14 skipping event	SSP predicted histological subtype	Proteomics analysis	Proteomics class	Mutation detected	*EGFR*	*KRAS*
1	Performed	Not performed	No	No	No	No	SCLC	Performed	Not classified	No	No	No
2	Performed	Performed	No	No	No	No	SqCC	Performed	ST6	Yes	No	Yes
3	Performed	Performed	No	No	No	No	AC	Performed	ST6	Yes	No	Yes
4	Performed	Performed	No	No	No	No	NOS	Performed	Not classified	Yes	No	Yes
5	Performed	Not performed	No	No	No	No	SqCC	Performed	Not classified	No	No	No
6	Performed	Performed	No	No	No	No	AC	Performed	ST1	Yes	No	Yes
7	Performed	Not performed	No	No	No	No	SCLC	Performed	ST5	No	No	No
8	Performed	Performed	No	No	No	No	SqCC	Performed	ST6	No	No	No
9	Performed	Performed	No	No	No	Yes	NOS	Performed	Not classified	No	No	No
10	Performed	Not performed	No	No	No	No	AC	Performed	ST1	Yes	Yes	No
11	Performed	Not performed	No	No	No	No	SqCC	Performed	ST3	No	No	No
12	Performed	Not performed	No	No	No	No	AC	Performed	ST3	No	No	No
13	Performed	Performed	No	No	No	No	AC	Performed	ST3	Yes	No	Yes
14	Performed	Not performed	No	No	No	No	SCLC	Performed	ST5	No	No	No
15	Performed	Performed	No	No	No	No	SCLC	Performed	ST5	No	No	No
16	Performed	Not performed	No	No	No	No	AC	Performed	Not classified	No	No	No
17	Performed	Performed	No	No	No	No	AC	Performed	ST3	No	No	No
18	Performed	Not performed	No	No	No	No	SCLC	Performed	Not classified	No	No	No
19	Performed	Performed	No	No	No	No	SqCC	Performed	ST6	No	No	No
20	Performed	Not performed	No	No	No	No	SCLC	Performed	ST3	No	No	No
21	Performed	Performed	No	No	No	No	AC	Performed	ST2	No	No	No
22	Performed	Not performed	No	No	No	No	SqCC	Performed	ST6	No	No	No
23	Performed	Not performed	No	No	No	No	SqCC	Performed	ST1	No	No	No
24	Performed	Not performed	No	No	No	No	AC	Performed	Not classified	Yes	No	Yes
25	Performed	Performed	No	No	No	No	SqCC	Performed	ST6	Yes	No	Yes
26	Performed	Not performed	No	No	No	No	AC	Performed	ST1	No	No	No
27	Performed	Performed	No	No	No	No	SCLC	Performed	ST5	No	No	No
28	Performed	Not performed	No	No	No	No	AC	Performed	ST3	No	No	No
29	Performed	Performed	No	No	No	No	AC	Performed	ST6	No	No	No
30	Performed	Performed	No	No	No	No	SqCC	Performed	ST6	No	No	No
31	Performed	Not performed	No	No	No	No	SqCC	Performed	ST6	No	No	No
32	Performed	Performed	No	No	No	No	AC	Performed	ST6	Yes	No	Yes
33	Performed	Performed	No	No	No	No	AC	Performed	ST3	Yes	Yes	No
34	Performed	Performed	No	No	No	No	AC	Performed	Unclassified	No	No	No
35	Performed	Not performed	No	No	No	No	SqCC	Performed	Unclassified	No	No	No
36	Performed	Not performed	No	No	No	No	SqCC	Performed	Not classified	No	No	No
37	Performed	Performed	No	No	No	No	AC	Performed		No	No	No
38	Performed	Performed	No	No	No	No	SqCC	Performed	Not classified	No	No	No
39	Performed	Not performed	No	No	No	No	AC	Performed	Not classified	Yes	Yes	No
40	Performed	Performed	No	No	No	No	SCLC	Performed	ST5	No	No	No
41	Performed	Performed	No	No	No	No	AC	Performed	ST6	No	No	No
42	Performed	Performed	No	No	No	No	AC	Performed	ST2	No	No	No
43	Performed	Performed	No	No	No	No	AC	Performed	ST1	Yes	No	Yes
44	Performed	Performed	No	No	No	No	AC	Performed	ST3	Yes	Yes	No
45	Performed	Performed	No	No	No	No	Excluded	Not performed	NA	No	No	No

Supplementary table 3. Clinical management, histopathological and treatment predictive analysis and study specific analysis. Abbreviations: SCLC, small cell lung cancer; SqCC, squamous cell carcinoma; AC, adenomcarcinoma, NOS, not-otherwise-specified; NGS, next-generation sequencing; FFPE, formalin fixed praffin embedded

Sample ID	Gene ID														
	AKT1	*BRAF*	*EGFR*	*ERBB2*	*FOXL2*	*GNA11*	*GNAQ*	*KIT*	*KRAS*	*MET*	*NRAS*	*PDGFRA*	*PIK3CA*	*RET*	*TP53*
1	NA	NA	NA	Ile655Val	NA	NA	NA	NA	NA	NA	NA	NA	NA	NA	Gly245Arg
2	NA	NA	NA	NA	NA	NA	NA	NA	Gly12Asp	NA	Thr124Ala	NA	NA	NA	Pro278Thr
3	NA	NA	NA	NA	NA	NA	NA	Met541Leu	Gly12Cys	NA	NA	NA	NA	NA	NA
4	NA	NA	NA	Ile655Val	NA	NA	NA	NA	Gly12Cys	NA	NA	NA	NA	NA	NA
5	NA	NA	NA	Ile655Val	NA	NA	NA	Met541Leu	NA	NA	NA	NA	NA	NA	NoAnnotatedProtChange
6	NA	NA	NA	NA	NA	NA	NA	NA	Gly12Asp	NA	NA	NA	NA	NA	NA
7	NA	NA	NA	NA	NA	NA	NA	NA	NA	NA	NA	NA	NA	NA	Tyr126Ter
8	NA	NA	NA	NA	NA	NA	NA	NA	NA	NA	NA	NA	NA	NA	Tyr234Cys
9	NA	NA	NA	Ile655Val	NA	NA	NA	NA	NA	NA	NA	NA	NA	NA	Pro85LeufsTer38
10	NA	NA	SerVal768IleLeu	Ile655Val	NA	NA	NA	NA	NA	NA	NA	NA	NA	NA	NA
11	NA	NA	NA	Ile655Val	NA	NA	NA	NA	NA	NA	NA	NA	NA	NA	NA
12	NA	NA	NA	Ile655Val	NA	NA	NA	Met541Leu	NA	NA	NA	NA	NA	NA	NA
13	NA	NA	NA	Ile655Val	NA	NA	NA	NA	Gly12Arg	NA	NA	NA	NA	NA	Arg158Leu
14	NA	NA	NA	NA	NA	NA	NA	NA	NA	NA	NA	NA	NA	NA	Arg273Leu
15	NA	NA	NA	NA	NA	NA	NA	NA	NA	NA	NA	NA	NA	NA	Cys176Ter
16	NA	NA	NA	NA	NA	NA	NA	NA	NA	NA	NA	NA	NA	NA	NoAnnotatedProtChange
17	NA	NA	NA	Ile655Val	NA	NA	NA	NA	NA	NA	NA	NA	NA	NA	Val274Phe
18	NA	NA	NA	NA	NA	NA	NA	NA	NA	NA	NA	NA	NA	NA	Glu298Ter
19	NA	NA	NA	Ile655Val	NA	NA	NA	NA	NA	NA	NA	NA	NA	NA	His179Tyr
20	NA	NA	NA	NA	NA	NA	NA	NA	NA	NA	NA	NA	NA	NA	Tyr236Cys
21	NA	NA	NA	Ile655Val	NA	NA	NA	Met541Leu	NA	NA	NA	NA	NA	NA	Ser241Phe
22	NA	NA	NA	NA	NA	NA	NA	Met541Leu	NA	NA	NA	NA	NA	NA	Ser303AlafsTer42:Pro301Arg
23	NA	NA	NA	Ile655Val	NA	NA	NA	NA	NA	NA	NA	NA	NA	NA	NA
25	NA	NA	NA	NA	NA	NA	NA	NA	Gly12Cys	NA	NA	NA	Arg524Lys	NA	NA
26	NA	NA	NA	NA	NA	NA	NA	NA	NA	NA	NA	NA	NA	NA	NA
27	NA	NA	NA	NA	NA	NA	NA	NA	NA	NA	NA	NA	NA	NA	Ser106Arg
28	NA	NA	NA	NA	NA	NA	NA	NA	NA	NA	NA	NA	NA	NA	Pro177Arg
29	NA	NA	NA	Ile655Val	NA	NA	NA	NA	NA	NA	NA	NA	NA	NA	NA
30	NA	NA	NA	Ile655Val	NA	NA	NA	Met541Leu	NA	NA	NA	NA	NA	NA	NoAnnotatedProtChange
31	NA	NA	NA	Ile655Val	NA	NA	NA	NA	NA	NA	NA	NA	NA	NA	Ser127Tyr
32	NA	NA	NA	NA	NA	NA	NA	Met541Leu	Gly12Phe	NA	NA	NA	NA	NA	NA
33	NA	NA	Leu858Arg	NA	NA	NA	NA	NA	NA	NA	NA	NA	NA	NA	Lys132Asn
34	NA	NA	NA	NA	NA	NA	NA	Met541Leu	NA	NA	NA	NA	NA	NA	NA
37	NA	NA	NA	NA	NA	NA	NA	NA	NA	NA	NA	NA	NA	NA	NA
38	NA	NA	NA	NA	NA	NA	NA	Met541Leu	NA	NA	NA	NA	NA	NA	Pro153AlafsTer28
39	NA	NA	Leu858Arg	NA	NA	NA	NA	NA	NA	NA	NA	NA	NA	NA	Gly244Cys
40	NA	NA	NA	NA	NA	NA	NA	NA	NA	NA	NA	NA	NA	NA	Phe270Cys
41	NA	NA	NA	NA	NA	NA	NA	NA	NA	NA	NA	NA	NA	NA	NA
42	NA	NA	NA	NA	NA	NA	NA	NA	NA	NA	NA	NA	NA	NA	NA
43	NA	NA	NA	NA	NA	NA	NA	Met541Leu	Gly12Asp	NA	NA	NA	NA	NA	Glu258Gly
24	NA	NA	NA	NA	NA	NA	NA	NA	Gly12Asp	NA	NA	NA	NA	NA	NA
35	NA	NA	NA	Ile655Val	NA	NA	NA	NA	NA	NA	NA	NA	NA	NA	NA
36	NA	NA	NA	Ile655Val	NA	NA	NA	NA	NA	NA	NA	NA	NA	NA	Arg249Ser
44	NA	NA	Glu746_Ala750del	NA	NA	NA	NA	NA	NA	NA	NA	NA	NA	NA	NA
45	NA	NA	NA	NA	NA	NA	NA	NA	NA	NA	NA	NA	NA	NA	NA

Supplementary table 4. Detected variants using the Illumina TST-15 platform. Predicted protein alterations in detected variant changes.

Probe Name	Accession #	Class Name	Sample ID																					
			1	2	3	4	7	8	9	10	11	12	13	14	15	16	17	18	19	20	21	22	24	25
ALK_3P-1	NG_009445.1	Endogenous	7	9	4	8	18	4	8	3	2	7	4	64	20	3	4	4	17	26	3	6	7	10
ALK_3P-2	NG_009445.1	Endogenous	9	11	11	9	17	8	16	2	10	2	12	42	9	7	7	2	31	19	4	13	2	7
ALK_3P-3	NG_009445.1	Endogenous	19	14	12	16	38	16	18	18	10	9	12	85	33	13	27	15	30	34	15	15	15	10
ALK_3P-4	NG_009445.1	Endogenous	23	12	12	13	25	15	7	10	11	5	7	39	22	4	16	12	24	25	12	17	14	15
ALK_5P-1	NG_009445.1	Endogenous	56	26	4	5	115	16	17	8	10	11	7	199	102	6	11	3	66	123	9	12	3	6
ALK_5P-2	NG_009445.1	Endogenous	151	140	132	118	262	155	166	77	120	55	162	232	97	73	182	21	193	225	113	203	29	71
ALK_5P-3	NG_009445.1	Endogenous	21	12	8	3	47	5	2	1	6	5	1	89	35	5	10	3	54	50	5	5	4	7
ALK_5P-4	NG_009445.1	Endogenous	34	25	44	37	43	21	35	29	31	25	27	74	30	36	35	22	56	59	19	45	19	24
ANPEP	NM_001150.1	Endogenous	153	166	125	277	205	176	1518	69	397	359	443	107	71	320	446	41	104	151	145	186	66	257
ATP1B1	NM_001001787.1	Endogenous	822	2616	10958	1342	1911	2603	632	2156	868	1090	672	7228	552	627	2034	167	2854	1252	2519	10662	56	1157
ATP8A1	NM_006095.2	Endogenous	431	49	167	427	1422	43	258	190	564	161	106	3874	282	497	196	29	60	477	313	130	31	126
AURKA	NM_003600.2	Endogenous	483	37	94	80	601	118	179	43	35	21	283	337	228	89	144	48	283	181	532	149	25	86
BLM	NM_000057.2	Endogenous	526	21	34	45	363	163	74	21	24	39	109	105	585	37	171	20	137	133	268	67	10	83
BRCA1	NM_007305.2	Endogenous	859	108	35	53	625	111	65	33	46	29	166	339	343	91	125	37	181	180	394	95	15	59
C1orf116	NM_023938.4	Endogenous	92	230	502	450	518	272	9	489	794	133	111	190	20	680	89	5	66	11	125	282	128	219
CCDC6-RET_C1:R12	CCDC6-RET_C1:R12	Endogenous	15	9	20	6	20	17	9	7	14	20	10	17	14	15	22	16	18	18	17	22	19	12
CCNA2	NM_001237.2	Endogenous	607	39	104	86	794	252	265	65	33	26	829	663	566	119	232	84	829	293	969	359	24	176
CCNE1	NM_001238.1	Endogenous	663	21	52	91	573	148	79	17	27	12	206	643	249	31	307	21	680	204	274	120	20	70
CD20	NM_152866.2	Endogenous	62	14	12	58	72	36	47	102	252	28	144	95	26	88	267	10	44	153	1954	119	13	57
CD247	NM_198053.1	Endogenous	207	55	49	253	239	76	540	129	264	165	251	230	35	205	198	30	85	472	637	225	34	140
CD3D	NM_000732.4	Endogenous	174	82	44	249	247	69	608	106	255	162	377	269	25	172	351	5	114	792	751	332	25	167
CD3E	NM_000733.2	Endogenous	283	105	67	493	492	120	1173	220	453	405	574	403	57	359	549	23	169	1340	1523	621	56	340
CD3G	NM_000073.2	Endogenous	44	18	11	91	118	22	244	46	83	91	124	110	11	78	122	5	38	277	250	158	12	66
CD4	NM_000616.4	Endogenous	322	867	261	990	1474	411	2258	544	1533	311	1376	608	79	853	1825	19	196	1808	536	1244	41	405
CD56	NM_000615.5	Endogenous	1388	37	23	18	904	49	23	16	29	26	20	1255	1485	18	13	105	26	191	18	28	7	25
CD68	NM_001251.2	Endogenous	553	328	1587	4006	1810	1297	4645	1029	3913	245	2826	901	209	625	3021	35	547	3811	1047	1719	179	448
CD69	NM_001781.1	Endogenous	105	42	12	148	95	42	274	70	118	293	184	62	24	115	170	4	38	291	431	176	18	184
CD74-NRG1_C6:N6	CD74-NRG1_C6:N6	Endogenous	9	8	9	11	12	9	6	4	8	8	5	14	11	8	10	8	15	23	13	10	14	8
CD74-NRG1_C8:N6	CD74-NRG1_C8:N6	Endogenous	8	13	8	4	8	6	4	4	5	6	8	11	12	9	9	1	11	9	4	12	3	8
CD74-ROS1_C6:R32	CD74-ROS1_C6:R32	Endogenous	3	1	4	1	3	2	1	1	3	1	6	7	7	3	8	4	6	8	6	3	3	9
CD74-ROS1_C6:R34	CD74-ROS1_C6:R34	Endogenous	16	17	40	14	24	25	17	20	13	8	11	14	10	8	17	6	58	11	13	39	7	15
CD74_8:NTRK1_12	CD74_8:NTRK1_12	Endogenous	89	83	68	56	118	86	102	40	70	41	94	52	31	44	109	23	93	93	63	122	15	54
CD8A	NM_001768.5	Endogenous	431	121	42	343	527	84	2172	189	331	339	401	370	45	195	275	9	187	1549	160	424	15	178
CDKN3	NM_005192.3	Endogenous	700	11	109	40	665	218	204	47	23	22	309	596	382	70	335	98	447	220	627	165	27	200
CHGA	NM_001275.3	Endogenous	2115	92	14	15	2089	20	7	19	20	18	13	14897	1021	10	22	355	18	1492	365	253	19	22
COL4A3	NM_000091.4	Endogenous	183	5	20	191	344	12	12	82	97	9	24	16	8	177	62	4	11	19	64	10	6	18
CTLA4	NM_005214.3	Endogenous	18	5	21	35	42	25	135	31	25	12	137	30	13	78	203	4	54	200	84	161	4	76
CYP2B6	NM_000767.4	Endogenous	166	654	75	304	94	2113	7	279	408	152	24	43	12	826	826	22	52	40	98	67	89	62
DLC1	NM_006094.3	Endogenous	329	7	151	653	891	181	77	340	886	259	187	423	137	296	258	7	48	231	14	170	16	316
DOCK9	NM_001130048.1	Endogenous	703	500	430	895	868	372	364	727	900	597	359	764	1338	354	677	53	242	325	301	710	48	1108
DPP4	NM_001935.3	Endogenous	85	202	317	188	303	48	490	979	229	146	446	75	10	1598	86	11	58	159	1416	69	69	80
EDN3	NM_000114.2	Endogenous	22	9	24	28	15	10	11	4	29	17	10	8	6	5	14	6	22	14	12	16	7	16
EML4-ALK_E13:A20	EML4-ALK_E13:A20	Endogenous	56	56	66	54	66	54	61	33	47	22	49	30	21	24	47	10	61	57	21	67	10	24
EML4-ALK_E13:ins69A20	EML4-ALK_E13:ins69A20	Endogenous	14	13	12	9	13	17	14	15	16	10	12	10	13	12	16	5	14	17	11	16	10	8
EML4-ALK_E14:del12A20	EML4-ALK_E14:del12A20	Endogenous	5	5	2	2	7	8	8	5	3	6	6	9	7	9	4	7	9	10	4	6	3	9
EML4-ALK_E14:ins11del49A20	EML4-ALK_E14:ins11del49A20	Endogenous	12	14	20	9	22	9	8	16	6	3	8	10	11	5	6	3	23	10	5	10	7	4
EML4-ALK_E15:A20	EML4-ALK_E15:A20	Endogenous	22	19	15	10	15	16	2	15	10	16	12	27	15	12	19	16	19	23	14	11	18	17
EML4-ALK_E17:ins30A20	EML4-ALK_E17:ins30A20	Endogenous	7	10	16	4	8	13	9	5	14	6	15	6	9	5	14	4	15	12	6	22	6	3
EML4-ALK_E18:A20	EML4-ALK_E18:A20	Endogenous	18	21	10	16	21	13	7	21	18	19	17	9	14	7	29	11	25	23	9	21	12	12
EML4-ALK_E20:A20	EML4-ALK_E20:A20	Endogenous	7	3	7	5	11	5	6	11	4	3	7	7	3	4	8	3	13	9	7	9	5	6
EML4-ALK_E2:A20	EML4-ALK_E2:A20	Endogenous	4	8	4	5	7	5	4	7	3	5	1	5	8	5	2	2	30	5	3	15	5	4
EML4-ALK_E2:ins117A20	EML4-ALK_E2:ins117A20	Endogenous	4	4	13	4	4	16	10	10	5	4	5	6	5	8	8	3	9	9	8	16	5	7
EML4-ALK_E6a:A20	EML4-ALK_E6a:A20	Endogenous	10	10	6	1	10	10	7	10	6	5	4	7	10	4	13	5	15	4	5	13	13	5
EML4-ALK_E6b:A20	EML4-ALK_E6b:A20	Endogenous	6	8	5	5	5	11	6	9	5	2	4	5	5	2	9	1	18	5	2	11	3	9
ERN1	NM_001433.2	Endogenous	383	184	250	380	290	206	285	266	310	269	175	624	169	232	314	36	216	299	301	228	37	230
EZR-ROS1_E10:R34	EZR-ROS1_E10:R34	Endogenous	6	11	11	8	13	3	12	9	3	7	7	12	19	11	18	11	16	20	3	6	6	13
FA2H	NM_024306.2	Endogenous	156	307	689	399	958	54	62	281	27	55	21	233	118	27	375	12	59	76	403	26	10	67
FAM64A	NM_019013.1	Endogenous	11	16	38	11	16	27	11	8	13	6	15	18	7	1	15	2	20	14	13	31	3	5
FANCI	NM_001113378.1	Endogenous	964	59	70	48	1118	429	161	41	34	42	161	236	1230	71	236	40	281	215	381	124	13	130
FOSL2	NM_005253.3	Endogenous	838	877	4372	1700	1067	1731	798	313	540	1074	1383	770	480	910	1307	153	3139	908	2294	2664	130	1408
FOXP3	NM_014009.3	Endogenous	15	18	25	34	45	22	95	26	17	18	66	34	19	39	103	9	43	46	54	91	10	41
GOPC-ROS1_G4:R36	GOPC-ROS1_G4:R36	Endogenous	2	2	4	4	9	8	6	8	1	3	7	7	6	3	4	1	6	10	5	7	4	2
GOPC-ROS1_G7:R35	GOPC-ROS1_G7:R35	Endogenous	13	16	19	9	18	9	11	7	13	7	7	11	10	3	11	3	14	16	11	20	4	12
GOPC-ROS1_G8:R35	GOPC-ROS1_G8:R35	Endogenous	19	7	19	8	24	25	14	8	12	5	14	18	13	8	8	5	23	12	10	19	5	6
GPR116	NM_015234.3	Endogenous	673	76	109	1733	1541	252	66	1512	1987	249	195	680	210	1103	595	11	78	252	17	249	33	436
HEXIM1	NM_006460.2	Endogenous	660	625	394	909	850	779	803	491	546	670	1010	901	474	492	615	149	1135	517	1713	1076	59	678
HIF1A	NM_001530.2	Endogenous	2027	804	2382	1257	2274	2887	3597	821	711	1386	3481	2635	938	912	2253	327	3034	1467	745	1628	155	1745
HOPX	NM_139212.3	Endogenous	910	440	9416	1832	3063	1035	166	2089	2711	155	442	687	123	2576	696	37	301	556	78	393	1906	217
HSD17B6	NM_003725.2	Endogenous	183	29	56	280	393	24	12	142	853	13	40	250	46	7	301	9	44	57	11	25	3	8
IFT57	NM_018010.2	Endogenous	2664	5453	243	724	1128	384	491	821	826	535	362	1659	300	1671	446	115	793	508	444	1058	51	386
IKBKAP	NM_003640.3	Endogenous	544	574	167	232	1164	236	240	186	222	236	204	734	236	176	278	44	505	389	296	319	19	187
KIF14	NM_014875.2	Endogenous	134	7	20	25	234	48	51	18	8	11	59	264	114	27	39	26	74	104	127	57	14	42
KIF5B-ALK_K15:A20	KIF5B-ALK_K15:A20	Endogenous	9	11	6	3	1	6	3	6	6	8	5	6	4	6	6	4	8	8	4	8	7	11
KIF5B-ALK_K17:A20	KIF5B-ALK_K17:A20	Endogenous	33	27	25	24	25	25	35	34	34	35	22	32	46	36	32	43	42	43	25	27	31	24
KIF5B-ALK_K24:A20	KIF5B-ALK_K24:A20	Endogenous	6	7	6	2	4	2	3	2	7	3	4	6	4	1	2	5	6	2	5	7	1	3
KIF5B-RET_K15:R11	KIF5B-RET_K15:R11	Endogenous	14	17	18	13	25	17	14	7	14	9	17	15	14	8	15	4	21	14	12	23	8	11
KIF5B-RET_K15:R12	KIF5B-RET_K15:R12	Endogenous	8	8	16	6	6	4	6	10	12	6	7	7	5	6	6	4	29	8	9	7	5	8
KIF5B-RET_K16:R12	KIF5B-RET_K16:R12	Endogenous	23	33	27	24	33	26	26	31	21	24	16	27	41	30	34	18	49	44	25	36	27	24
KIF5B-RET_K22:R12	KIF5B-RET_K22:R12	Endogenous	11	3	9	6	10	5	4	6	5	4	11	4	6	5	3	4	14	4	8	4	2	7
KIF5B-RET_K23:R12	KIF5B-RET_K23:R12	Endogenous	14	18	23	12	28	10	10	6	13	5	15	18	6	5	17	6	19	13	9	24	5	4
KIF5B-RET_K24:R11	KIF5B-RET_K24:R11	Endogenous	4	7	13	4	9	11	6	10	6	3	13	4	1	4	9	5	9	4	8	3	4	6
KIF5B-RET_K24:R8	KIF5B-RET_K24:R8	Endogenous	21	13	17	16	14	18	18	16	15	23	15	14	21	16	18	15	23	28	20	30	16	17
KRT16	NM_005557.3	Endogenous	38	394	188	48	44	270	37	33	78	16	44	24	22	20	95	10	1368	32	15	3160	15	908
KRT40	NM_182497.3	Endogenous	15	156	5	9	6	38	3	6	10	34	4	6	3	3	132	4	24	10	13	14	3	3
KRT5	NM_000424.2	Endogenous	189	9164	58	172	1132	43519	111	107	545	6567	314	178	262	130	31	60	79216	138	35	62688	15	19556
KRT6A	NM_005554.3	Endogenous	17	1564	752	40	5	1569	6	1	23	27	452	12	26	5	185	12	6135	10	2	4245	17	1012
L1CAM	NM_024003.2	Endogenous	1191	8	11	22	1922	15	20	15	28	69	94	1599	225	24	26	214	11	802	16	19	9	72
LOC81691	NM_030941.2	Endogenous	607	88	22	19	345	16	34	23	19	22	24	183	463	46	44	11	75	92	63	36	6	34
LRIG3-ROS1_L16:R35	LRIG3-ROS1_L16:R35	Endogenous	3	7	8	2	7	9	2	6	8	5	3	4	11	4	7	3	14	10	4	10	1	7
MB	NM_005368.2	Endogenous	82	1171	34	15	38	35	8	23	14	104	56	245	49	38	244	15	70	28	45	63	6	71
MBIP	NM_001144891.1	Endogenous	1323	118	511	314	536	218	179	507	462	106	257	1905	820	366	309	340	290	365	182	163	51	101
MDM2	NM_001145337.1	Endogenous	230	107	43	219	151	122	65	49	61	100	60	203	115	90	81	24	205	136	109	125	17	227
METex13-15	ENST00000454623.1	Endogenous	252	160	367	277	52	408	736	100	122	125	293	785	49	148	241	44	214	60	106	362	31	101
METex14	NM_001127500.1	Endogenous	1071	451	1119	798	184	1202	44	285	406	276	767	2491	144	629	775	93	541	137	313	962	65	315
METex20-21	NM_000245.2	Endogenous	1106	624	1750	1231	287	1755	994	447	558	471	1281	3871	178	780	1317	174	873	206	1839	1600	144	532
METex3-4	NM_000245.2	Endogenous	1164	503	1295	973	229	1433	862	314	498	274	970	2368	183	539	1034	172	664	203	312	1046	123	341
MKI67 (Ki67)	NM_002417.2	Endogenous	1471	16	143	69	1264	282	479	48	45	11	693	656	1675	127	362	71	561	670	881	449	28	191
MLANA	NM_005511.1	Endogenous	5	11	15	12	9	13	4	12	12	12	7	15	17	14	11	13	16	11	14	14	11	10
MPRIP_23:NTRK1_12	MPRIP_23:NTRK1_12	Endogenous	5	8	9	3	8	14	6	4	7	6	7	5	5	2	8	3	13	3	9	5	2	9
MYT1L	NM_015025.2	Endogenous	152	2	4	3	891	3	2	2	3	4	2	11	7	8	6	5	5	9	5	10	3	5
NKX2-1	NM_003317.3	Endogenous	1690	99	502	373	880	35	146	1101	814	43	393	2299	721	556	361	212	17	473	7	45	127	34
NPM1-ALK_N4:A20	NPM1-ALK_N4:A20	Endogenous	10	5	4	4	5	6	3	8	8	4	2	4	5	5	2	5	12	4	4	3	7	4
NRG1_3p_1	NM_013958.3	Endogenous	122	47	44	63	55	41	38	36	48	43	44	176	64	92	249	33	93	98	27	60	33	40
NRG1_3p_2	NM_013962.2	Endogenous	85	32	27	38	19	34	28	9	24	29	15	161	23	73	201	6	54	62	11	40	5	17
NRG1_5p_1	NM_013960.3	Endogenous	298	43	30	92	59	75	59	11	50	117	20	568	121	243	524	29	176	98	31	111	6	76
NRG1_5p_2	NM_004495.3	Endogenous	224	45	37	73	53	69	57	19	51	88	21	481	72	181	355	27	174	76	19	68	3	50
NTRK1_3P1	NM_002529.3	Endogenous	12	10	5	8	10	5	5	11	8	8	10	66	15	11	17	11	22	13	6	15	8	13
NTRK1_3P2	NM_002529.3	Endogenous	16	17	16	3	24	19	8	13	18	9	17	143	6	12	18	5	19	19	8	18	1	14
NTRK1_3P3	NM_002529.3	Endogenous	10	7	16	6	24	17	6	7	18	6	15	63	7	12	10	2	13	16	8	14	2	6
NTRK1_3P4	NM_002529.3	Endogenous	15	6	6	15	23	14	9	13	17	16	17	142	14	15	12	4	10	18	3	13	2	18
NTRK1_5P1	NM_002529.3	Endogenous	6	8	9	10	9	11	6	11	5	12	8	46	9	9	7	6	17	11	9	13	8	19
NTRK1_5P2	NM_002529.3	Endogenous	4	1	1	1	4	3	2	3	7	4	2	51	1	10	3	1	7	5	1	5	4	7
NTRK1_5P3	NM_002529.3	Endogenous	2	8	6	6	9	6	7	9	11	8	16	111	3	11	13	5	13	15	6	9	3	10
NTRK1_5P4	NM_002529.3	Endogenous	30	28	25	20	28	24	17	25	21	12	16	132	12	18	32	24	26	28	18	33	12	22
Napsin A (NAPSA)	NM_004851.1	Endogenous	2501	21	27221	5110	6607	251	30	26135	12952	12	2028	178	15	5706	412	9	30	114	22	500	2181	13
PD1	NM_005018.1	Endogenous	11	13	6	14	27	12	155	14	18	15	83	24	5	13	46	4	19	130	37	60	3	24
PDL1	NM_014143.3	Endogenous	29	29	17	81	47	23	68	26	37	24	68	44	26	24	49	22	86	107	19	79	15	22
PRC1	NM_199413.1	Endogenous	2021	40	114	70	1730	416	240	48	35	33	313	487	2088	92	245	65	436	228	471	144	21	169
RET_3P-1	NM_020630.4	Endogenous	560	48	5	5	83	6	7	7	10	7	11	1412	332	7	14	31	7	143	14	25	2	30
RET_3P-2	NM_020630.4	Endogenous	460	94	28	31	119	32	16	29	27	23	30	1445	246	24	22	34	33	150	20	62	12	41
RET_3P-3	NM_020630.4	Endogenous	732	99	4	9	153	12	10	16	21	27	22	2065	354	23	27	27	12	177	13	60	1	42
RET_3P-4	NM_020630.4	Endogenous	268	58	22	13	63	19	18	7	27	13	15	706	160	13	30	9	24	71	11	41	9	20
RET_5P-1	NM_020630.4	Endogenous	626	61	22	11	127	16	15	15	21	13	16	1350	261	10	19	10	23	132	17	54	1	32
RET_5P-2	NM_020630.4	Endogenous	907	90	17	24	174	7	16	9	20	15	16	1966	354	20	23	30	18	183	12	46	7	33
RET_5P-3	NM_020630.4	Endogenous	444	66	9	7	107	8	8	11	19	17	21	1459	154	13	19	24	11	103	11	29	1	17
RET_5P-4	NM_020630.4	Endogenous	635	49	16	10	105	14	8	15	16	14	25	1553	307	20	14	37	23	167	14	37	2	26
ROS1_3P-1	NM_002944.2	Endogenous	132	14	32	306	314	153	28	421	522	12	15	23	6	170	109	4	39	21	5	77	40	11
ROS1_3P-2	NM_002944.2	Endogenous	152	12	26	333	314	169	28	506	531	16	23	26	13	199	133	9	34	33	14	51	32	13
ROS1_3P-3	NM_002944.2	Endogenous	114	13	26	241	275	114	22	342	433	23	33	24	18	158	111	14	26	34	17	44	43	17
ROS1_3P-4	NM_002944.2	Endogenous	114	11	34	241	282	144	19	345	408	6	11	16	4	150	100	1	14	15	3	47	19	7
ROS1_5P-1	NM_002944.2	Endogenous	207	10	95	557	785	488	66	1211	1208	8	87	20	6	317	347	1	141	30	7	151	92	15
ROS1_5P-2	NM_002944.2	Endogenous	142	10	29	289	356	235	38	629	634	14	58	22	20	298	233	5	54	27	8	105	78	13
ROS1_5P-3	NM_002944.2	Endogenous	214	1	70	540	670	413	54	1124	1050	11	86	34	8	351	353	4	112	22	8	124	70	14
ROS1_5P-4	NM_002944.2	Endogenous	126	10	26	207	371	220	25	533	463	7	31	12	9	181	123	6	21	17	6	93	11	5
RRM2	NM_001034.1	Endogenous	2271	99	524	140	2767	511	698	110	60	34	1242	838	1830	169	612	118	819	460	2555	615	35	334
SDC4-ROS1_S2:R32	SDC4-ROS1_S2:R32	Endogenous	21	16	36	14	35	27	17	17	27	6	20	17	10	5	13	4	31	22	19	23	5	13
SDC4-ROS1_S4:R32	SDC4-ROS1_S4:R32	Endogenous	11	10	11	10	9	8	6	13	4	6	15	8	10	7	7	5	15	8	8	3	7	11
SDC4-ROS1_S4:R34	SDC4-ROS1_S4:R34	Endogenous	17	17	15	22	20	20	14	14	15	33	20	24	26	21	27	21	25	23	17	27	24	24
SFTPB	NM_000542.3	Endogenous	7030	52	67013	14861	21126	1041	1059	17492	37311	12	20	1257	24	1233	3491	17	49	728	12	2885	1669	13
SFTPG	NM_205854.2	Endogenous	247	7	4009	2282	979	27	6	3171	1669	10	72	26	14	831	874	13	15	26	559	46	363	11
SLC34A2-ROS1_S13del2046:R32	SLC34A2-ROS1_S13del2046:R32	Endogenous	5	12	3	10	11	9	11	2	2	6	7	3	6	5	4	1	6	7	6	15	1	5
SLC34A2-ROS1_S4:R32	SLC34A2-ROS1_S4:R32	Endogenous	35	31	51	26	40	26	22	11	25	19	60	31	21	15	32	17	85	30	63	48	7	42
SLC34A2-ROS1_S4:R34	SLC34A2-ROS1_S4:R34	Endogenous	14	16	17	11	24	14	12	8	12	4	6	6	7	3	13	2	15	7	9	10	3	7
SLC35A5	NM_017945.2	Endogenous	669	404	246	249	689	252	192	146	259	203	184	789	422	126	304	48	160	252	236	331	16	180
STMN2	NM_007029.2	Endogenous	3321	15	20	21	2768	18	16	25	14	29	30	16	19	9	28	10	22	386	15	17	10	24
SYP	NM_003179.2	Endogenous	695	38	40	64	1214	33	33	35	82	38	35	1204	253	44	58	66	35	496	70	49	37	41
TFG-ALK_T5:A20	TFG-ALK_T5:A20	Endogenous	6	3	12	12	5	6	5	8	5	4	5	5	2	5	10	4	20	9	7	7	5	5
TFG-ALK_T5:A20	TFG-ALK_T5:A20	Endogenous	6	6	2	4	2	4	4	4	3	2	4	8	4	4	7	2	8	7	3	6	4	7
TFG-ALK_T6:A20	TFG-ALK_T6:A20	Endogenous	11	6	15	5	10	8	4	14	11	7	10	11	2	4	5	2	14	15	10	3	8	2
TP73L	NM_003722.4	Endogenous	56	816	9	77	51	2855	376	27	150	1289	73	36	48	25	566	7	4466	44	14	3839	12	470
TPM3-ROS1_T8:R35	TPM3-ROS1_T8:R35	Endogenous	7	4	11	6	10	6	7	4	8	4	10	8	7	4	9	8	14	12	7	14	8	7
TPSAB1	NM_003294.3	Endogenous	201	558	52	2037	709	1554	26	1707	5568	3367	2644	261	85	3434	1619	6	952	1307	65	1928	26	12214
TREM1	NM_001242589.1	Endogenous	152	59	363	568	30	61	143	81	567	177	173	349	49	338	449	52	55	112	55	50	137	74
TRIM14	NM_014788.2	Endogenous	685	386	130	375	472	185	947	243	418	235	480	1169	443	278	583	55	455	568	686	552	61	342
TRIM24-BRAF_T5:B8	TRIM24-BRAF_T5:B8	Endogenous	9	9	16	7	16	8	6	4	6	5	11	11	4	6	13	2	8	8	4	13	2	6
TRIM24-BRAF_T9:B9	TRIM24-BRAF_T9:B9	Endogenous	7	9	8	12	13	8	5	7	4	11	10	13	9	11	11	9	19	9	19	8	6	3
TTC37	NM_014639.3	Endogenous	709	661	956	725	1962	454	490	376	637	614	464	930	761	524	624	93	589	730	923	864	69	639
TTF-1	NM_007344.2	Endogenous	885	266	113	202	838	130	214	110	195	98	117	449	351	163	134	48	163	244	188	129	19	121
TYMS	NM_001071.1	Endogenous	8879	75	221	197	3514	505	912	122	81	83	757	2476	3446	437	506	512	591	2020	1253	751	51	579
UMPS	NM_000373.2	Endogenous	252	75	55	38	204	62	64	40	45	54	44	326	192	56	74	17	114	95	173	89	7	80
XPO1	NM_003400.3	Endogenous	2715	1203	1204	1276	3224	1225	1062	649	726	649	1446	2491	1503	1360	1524	209	2721	1305	2372	2113	134	927
ZMYND10	NM_015896.3	Endogenous	199	4712	16	205	51	19	19	27	106	253	59	231	46	367	18	68	95	122	14	225	5	39
ZNF236	NM_007345.3	Endogenous	874	218	192	228	439	178	248	123	222	122	127	520	533	162	212	44	201	401	302	184	8	147
ACTB	NM_001101.2	Housekeeping	82730	70209	63846	69938	119050	91356	104267	39696	62734	42969	92470	89796	30823	46842	88551	12976	82093	81730	69909	108255	12097	55135
CLTC	NM_004859.2	Housekeeping	2580	3035	3730	2229	4688	3737	4240	2607	1777	1278	2881	4737	975	2617	4149	337	3116	2281	2378	3591	242	1789
GAPDH	NM_002046.3	Housekeeping	12407	10304	17191	9800	14147	10386	11623	5074	4441	8262	21198	20151	9567	6161	14672	6015	44747	13501	33686	21512	2228	26357
GUSB	NM_000181.1	Housekeeping	974	539	498	677	1094	510	866	565	641	269	531	1274	566	405	658	141	472	645	649	625	36	364
HPRT1	NM_000194.1	Housekeeping	1064	322	367	339	1516	525	560	171	223	218	511	773	481	337	566	128	1218	483	513	650	106	370
MRPL19	NM_014763.3	Housekeeping	582	491	1012	571	884	559	635	283	374	339	452	937	295	458	570	113	1396	438	1014	836	54	521
OAZ1	NM_004152.2	Housekeeping	6848	8925	4408	5586	6934	5840	8511	3061	5649	4342	5302	11740	2942	4168	4624	1873	8210	6034	6103	6064	1055	3570
PGK1	NM_000291.2	Housekeeping	3379	1978	3825	3022	2676	3760	2986	1392	1363	1463	2949	3657	1031	1852	4013	1218	4265	3988	2215	4565	762	2534
POLR2A	NM_000937.2	Housekeeping	1590	1528	1190	1170	1642	1106	859	596	871	973	1855	1781	1091	657	1128	181	1552	1149	1680	2548	81	1066
PSMC4	NM_006503.2	Housekeeping	574	865	778	440	963	1863	704	290	300	357	441	1175	262	243	947	171	7416	501	1495	1381	44	566
PUM1	NM_001020658.1	Housekeeping	1304	810	491	712	1163	662	635	436	630	446	718	1834	1327	546	730	127	766	1012	638	767	73	591
RPLP0	NM_001002.3	Housekeeping	11	4	3	9	10	3	6	5	5	3	9	7	5	10	15	6	18	6	5	11	4	8
SF3A1	NM_001005409.1	Housekeeping	934	905	801	674	1402	805	697	440	592	668	584	1507	481	350	726	169	979	740	1063	804	77	665
TFRC	NM_003234.1	Housekeeping	1806	568	582	1008	1069	3539	672	458	1005	413	989	3276	3447	623	3294	575	3058	1304	4634	2110	217	1139
TUBB	NM_178014.2	Housekeeping	5971	1078	1987	2066	11746	5165	3550	1134	2168	1158	3305	7726	3706	1174	2356	763	4834	4682	4397	4109	331	2595
NEG_A	ERCC_00096.1	Negative	1	4	3	2	1	4	2	2	1	1	1	5	1	1	4	3	5	3	2	4	2	4
NEG_B	ERCC_00041.1	Negative	3	5	9	2	6	3	4	9	3	3	8	5	7	3	1	5	11	6	10	9	5	4
NEG_C	ERCC_00019.1	Negative	3	1	8	3	5	4	3	4	7	2	5	5	4	7	8	1	5	6	3	5	5	5
NEG_D	ERCC_00076.1	Negative	1	1	5	2	6	1	1	3	5	2	2	3	4	1	4	1	6	3	1	5	1	1
NEG_E	ERCC_00098.1	Negative	5	2	2	3	7	1	5	1	8	3	1	2	3	6	3	4	9	5	3	3	2	2
NEG_F	ERCC_00126.1	Negative	17	15	130	23	38	29	13	29	59	12	9	9	12	10	21	4	43	8	8	34	3	13
POS_A	ERCC_00034.1	Positive	27567	23405	20557	25932	24871	23092	27214	23706	25359	25954	24470	28107	34198	29348	29943	27489	34251	34476	31705	27538	28244	29513
POS_B	ERCC_00112.1	Positive	4399	3663	3187	3980	3896	3555	4231	3751	3807	4529	3742	4386	5400	4711	4552	4510	5238	5179	4768	4261	4523	4729
POS_C	ERCC_00002.1	Positive	1151	909	681	1011	953	797	1136	1025	1020	1272	984	1217	1558	1369	1281	1336	1445	1529	1364	1222	1289	1356
POS_D	ERCC_00092.1	Positive	197	171	106	183	146	145	156	149	149	194	164	210	224	215	199	220	228	222	213	171	188	210
POS_E	ERCC_00035.1	Positive	88	75	73	93	91	80	111	83	95	101	81	107	124	111	106	90	111	113	106	98	89	99
POS_F	ERCC_00117.1	Positive	37	28	30	36	35	37	30	32	31	36	32	33	54	36	29	32	42	36	28	30	40	26

Probe Name	Sample ID																						
	26	27	28	29	30	31	32	33	34	36	35	23	37	38	39	40	41	42	43	44	45	5	6
ALK_3P-1	7	3	5	95	3	59	7	12	6	4	2	10	11	19	5	6	10	4	3	7	4	4	6
ALK_3P-2	6	8	6	52	8	48	6	14	9	5	4	2	4	11	6	2	10	6	6	6	3	11	7
ALK_3P-3	14	22	12	105	12	98	22	21	17	15	6	23	13	35	18	17	15	15	18	14	12	18	18
ALK_3P-4	12	8	14	39	10	51	16	29	10	19	10	10	12	29	6	20	17	11	17	12	13	15	7
ALK_5P-1	12	11	13	9	16	253	6	24	16	9	4	19	11	11	11	13	13	11	16	12	5	22	24
ALK_5P-2	84	192	41	150	110	286	68	148	100	95	35	74	119	82	77	13	128	193	110	110	1	170	136
ALK_5P-3	3	8	9	6	2	107	8	22	10	8	6	10	3	4	7	4	6	12	6	7	4	27	6
ALK_5P-4	36	30	23	25	30	70	29	35	23	31	12	27	31	16	26	37	22	31	33	21	21	34	36
ANPEP	312	313	97	202	171	182	191	426	259	81	93	124	200	244	226	21	315	366	290	597	12	273	504
ATP1B1	1695	1948	1706	3816	2283	1812	2756	1169	2698	188	283	607	579	213	921	87	452	1584	393	1108	4	4482	1938
ATP8A1	228	298	31	97	428	84	162	430	92	367	72	87	87	99	117	30	239	87	158	157	3	119	228
AURKA	31	317	65	83	267	230	100	77	33	67	105	28	22	47	34	84	79	111	201	38	1	197	170
BLM	176	170	43	60	147	256	77	47	23	80	80	19	20	54	18	61	83	50	52	31	4	141	76
BRCA1	54	310	11	63	144	144	78	60	145	52	32	22	30	39	33	141	58	102	98	54	2	200	132
C1orf116	71	163	45	497	71	39	150	227	358	7	8	56	21	13	87	5	60	175	66	253	2	693	3261
CCDC6-RET_C1:R12	19	20	14	14	9	10	23	16	12	18	6	16	24	11	12	14	19	14	20	25	10	20	19
CCNA2	27	1005	57	159	339	528	144	125	41	116	102	28	31	82	33	177	186	184	403	69	3	960	158
CCNE1	18	599	29	86	97	244	111	69	34	33	57	8	20	48	13	327	52	50	81	42	3	211	101
CD20	38	16	23	96	41	68	80	48	17	3082	188	24	27	146	37	19	644	43	268	97	11	22	22
CD247	50	81	86	101	156	200	137	172	80	507	160	106	82	251	109	25	559	139	181	180	21	70	68
CD3D	60	39	53	166	220	224	135	235	100	910	188	102	91	298	154	14	1085	152	271	282	6	82	50
CD3E	109	66	87	263	363	398	236	385	169	1666	356	197	172	575	282	14	1508	261	378	443	14	164	95
CD3G	11	12	15	48	74	76	55	83	42	308	76	36	29	92	48	2	363	44	77	99	5	30	31
CD4	101	411	130	439	505	568	543	1630	1017	515	375	292	431	1214	336	11	1339	499	359	432	4	509	336
CD56	31	1639	11	21	35	36	33	21	25	28	16	14	79	13	47	195	27	20	27	30	4	44	13
CD68	230	1433	1036	2293	1280	1199	1020	3505	558	476	1366	403	623	2351	387	41	2723	1021	2352	574	2	1446	1555
CD69	38	46	38	141	108	118	93	203	77	605	127	40	187	166	106	10	1158	118	367	133	4	35	117
CD74-NRG1_C6:N6	10	14	10	9	11	11	12	9	9	11	6	10	12	8	10	11	16	12	15	12	16	9	14
CD74-NRG1_C8:N6	2	7	3	10	13	7	10	6	8	7	6	9	12	8	9	5	9	13	7	3	7	14	12
CD74-ROS1_C6:R32	7	8	2	9	4	6	3	7	3	1	4	1	8	5	4	7	3	9	6	10	5	6	8
CD74-ROS1_C6:R34	5	25	2	23	30	40	8	18	14	12	4	13	12	10	15	9	11	26	14	14	3	43	19
CD74_8:NTRK1_12	40	108	34	67	81	85	36	81	55	56	21	44	62	47	32	14	76	104	57	63	1	107	80
CD8A	114	35	67	146	476	278	238	229	190	314	252	176	96	390	210	15	1012	174	515	325	4	108	33
CDKN3	24	459	93	175	386	209	116	82	44	68	161	34	55	69	37	366	138	165	287	56	5	717	245
CHGA	13	3996	53	16	195	32	25	18	43	15	15	19	15	26	27	463	23	27	18	14	17	146	33
COL4A3	8	19	17	10	14	15	52	27	3	83	21	11	26	26	13	8	20	6	20	223	3	6	78
CTLA4	14	75	19	48	76	118	27	122	10	97	48	6	27	115	21	37	463	75	123	26	2	58	36
CYP2B6	85	48	37	177	63	156	137	793	329	133	74	255	87	14	144	6	63	118	113	217	5	22	103
DLC1	159	497	79	304	205	133	117	295	130	27	15	154	326	262	234	8	426	280	20	447	3	190	898
DOCK9	844	807	149	516	537	574	418	665	701	276	278	231	505	196	545	133	465	851	189	714	9	1103	1094
DPP4	466	60	67	1984	84	109	175	557	107	110	32	35	53	67	104	15	135	82	543	531	2	112	943
EDN3	688	14	10	7	8	17	14	10	13	38	9	8	6	4	6	7	11	10	5	15	1	14	10
EML4-ALK_E13:A20	30	66	18	37	63	64	29	50	29	36	17	24	52	24	23	8	43	59	43	28	5	57	55
EML4-ALK_E13:ins69A20	8	14	10	10	10	7	18	17	17	16	8	25	18	11	15	18	17	18	17	14	20	13	19
EML4-ALK_E14:del12A20	5	8	5	4	3	1	3	4	9	9	2	5	12	4	7	9	9	5	12	9	7	12	4
EML4-ALK_E14:ins11del49A20	12	12	9	12	11	16	9	11	12	5	9	4	10	9	8	5	5	9	11	8	6	21	11
EML4-ALK_E15:A20	11	21	19	10	14	12	19	14	10	14	13	25	25	20	19	21	15	16	18	7	9	21	22
EML4-ALK_E17:ins30A20	9	13	4	9	11	20	5	4	11	3	2	10	12	5	9	6	6	6	16	8	3	12	11
EML4-ALK_E18:A20	21	14	16	18	16	14	26	13	16	15	8	21	28	12	7	14	21	21	16	7	17	10	25
EML4-ALK_E20:A20	4	7	5	14	11	7	3	8	4	6	4	8	4	7	7	5	16	8	8	6	8	9	10
EML4-ALK_E2:A20	3	5	4	8	12	5	9	7	5	5	1	5	10	4	6	7	9	9	9	6	5	9	6
EML4-ALK_E2:ins117A20	3	7	8	6	16	16	7	3	8	2	3	8	5	7	2	4	10	5	9	9	5	13	8
EML4-ALK_E6a:A20	6	8	5	17	7	8	13	7	8	6	3	15	13	12	10	9	19	16	14	9	13	9	15
EML4-ALK_E6b:A20	6	7	8	29	11	6	8	5	5	4	4	9	5	8	5	8	19	13	12	7	4	8	8
ERN1	245	282	62	209	253	213	270	334	166	173	126	69	132	119	145	100	227	264	143	226	2	216	469
EZR-ROS1_E10:R34	9	12	8	13	12	13	12	14	15	7	6	11	7	8	9	6	12	14	6	8	8	15	10
FA2H	168	176	350	371	78	52	255	47	219	32	36	59	24	8	33	35	35	44	53	72	6	56	255
FAM64A	10	16	3	8	13	8	12	17	14	5	5	10	7	6	6	5	9	13	16	5	5	15	11
FANCI	20	351	55	91	375	332	80	59	137	86	47	16	28	79	27	209	117	87	107	48	4	323	109
FOSL2	480	1421	1609	1146	2249	1408	688	1235	1088	275	2634	272	705	638	771	123	1562	1527	1337	1416	8	6165	3928
FOXP3	11	27	12	44	35	69	12	84	12	68	37	14	19	75	13	16	190	45	31	28	13	46	33
GOPC-ROS1_G4:R36	6	4	2	7	5	5	5	7	6	4	2	2	2	5	7	1	5	6	3	6	2	8	4
GOPC-ROS1_G7:R35	6	10	5	13	14	11	12	11	10	5	7	6	11	6	17	6	9	14	14	11	4	15	9
GOPC-ROS1_G8:R35	7	17	7	15	18	27	9	9	5	3	5	6	8	4	5	11	8	11	12	6	3	12	14
GPR116	206	880	68	517	300	200	166	746	241	21	17	135	267	573	145	18	724	486	47	744	5	167	4333
HEXIM1	468	888	143	745	810	529	617	600	640	323	500	324	610	259	570	238	603	955	410	899	14	1010	1049
HIF1A	1028	3596	751	1748	3916	1488	1113	1326	782	622	924	650	2067	1110	1651	320	2482	3383	1577	1519	6	2866	2263
HOPX	170	541	257	647	251	418	746	4168	106	170	65	140	249	335	185	29	303	569	1185	677	15	3051	5447
HSD17B6	11	152	14	38	30	61	18	102	28	7	4	17	41	10	17	32	13	46	16	108	5	71	137
IFT57	419	829	89	619	1042	1615	909	518	4917	522	345	1078	657	164	839	51	385	849	706	1991	8	749	644
IKBKAP	226	503	95	229	326	439	207	200	628	177	145	144	140	103	208	99	187	269	165	196	4	632	269
KIF14	13	167	18	30	94	67	46	19	17	17	13	10	10	21	11	46	37	41	30	9	11	134	72
KIF5B-ALK_K15:A20	2	8	7	9	4	5	7	5	8	5	5	11	13	1	7	10	4	10	6	7	8	8	10
KIF5B-ALK_K17:A20	43	44	27	35	31	36	38	38	40	34	19	42	27	21	37	27	33	28	27	27	35	38	29
KIF5B-ALK_K24:A20	8	3	2	6	8	6	5	4	5	1	3	6	1	3	4	2	6	5	8	5	1	7	2
KIF5B-RET_K15:R11	13	21	7	11	16	27	16	14	10	6	3	13	15	10	11	3	12	30	14	17	7	29	12
KIF5B-RET_K15:R12	6	8	3	9	17	9	3	7	8	6	8	8	11	12	10	7	10	10	11	8	3	7	13
KIF5B-RET_K16:R12	34	44	37	32	29	32	47	38	37	23	13	43	49	41	36	60	49	50	49	30	53	41	51
KIF5B-RET_K22:R12	4	9	5	5	5	7	5	6	5	3	3	7	10	5	11	8	9	6	13	7	4	6	5
KIF5B-RET_K23:R12	10	17	6	13	20	12	10	16	10	8	7	10	14	10	11	4	13	12	13	7	8	23	14
KIF5B-RET_K24:R11	6	7	8	10	9	10	4	4	3	2	2	2	6	4	2	7	9	2	18	4	7	7	5
KIF5B-RET_K24:R8	19	18	8	15	12	14	15	15	27	15	14	20	21	18	21	26	18	19	17	13	19	13	20
KRT16	18	1013	70	153	932	1118	45	92	32	27	171	26	23	19	20	6	205	139	40	65	6	19605	214
KRT40	1	15	7	23	14	37	14	7	37	3	6	44	7	3	16	121	58	23	15	16	5	9	25
KRT5	1941	18581	66	9028	36848	68446	3354	316	8714	887	2539	2575	2113	1526	6489	37	7155	23428	799	3794	2	108134	1428
KRT6A	9	2357	48	600	3627	4983	149	14	22	8	239	85	24	13	33	12	1073	1691	15	6	5	21150	47
L1CAM	81	239	29	10	27	10	35	21	52	21	7	60	153	10	97	69	25	340	87	41	6	37	18
LOC81691	13	162	11	31	42	73	29	30	106	24	15	41	26	16	25	73	20	23	53	34	3	39	79
LRIG3-ROS1_L16:R35	8	12	6	5	9	10	11	6	5	5	4	10	7	13	7	7	9	14	9	14	10	11	9
MB	227	41	11	42	60	143	103	43	604	50	15	99	99	2	144	6	23	113	82	100	1	37	79
MBIP	83	239	83	296	292	151	81	574	139	82	76	50	96	67	109	99	95	184	397	374	4	437	1395
MDM2	89	175	33	146	197	108	230	104	183	79	63	61	49	57	72	94	89	150	49	149	5	138	259
METex13-15	92	111	92	185	148	158	188	37	259	12	49	69	103	12	107	11	47	204	1621	211	6	284	375
METex14	300	350	334	587	500	510	542	109	768	42	164	213	243	40	373	8	134	730	4875	650	7	794	1206
METex20-21	507	581	548	1023	773	745	772	140	1332	45	222	292	374	65	638	7	236	1149	7553	1018	5	1688	2240
METex3-4	336	384	457	662	591	546	549	139	882	58	203	257	288	64	459	30	148	852	6177	674	23	919	1462
MKI67 (Ki67)	13	1389	97	313	589	365	130	142	40	111	149	26	29	136	11	200	227	267	278	59	2	688	294
MLANA	10	10	14	6	11	9	14	13	9	6	13	10	15	7	6	14	11	7	16	9	9	17	18
MPRIP_23:NTRK1_12	7	5	13	10	11	9	6	13	10	4	3	13	8	8	7	11	6	9	16	9	13	8	10
MYT1L	5	94	3	8	9	11	2	2	6	7	3	4	3	1	4	37	6	3	9	9	4	7	6
NKX2-1	47	356	28	576	25	35	52	710	198	11	7	55	24	28	92	146	28	227	681	417	9	9	1554
NPM1-ALK_N4:A20	5	6	6	5	6	12	7	6	4	4	6	8	12	4	5	8	16	1	7	5	8	7	9
NRG1_3p_1	31	118	26	34	51	73	58	50	61	25	69	44	43	34	72	35	80	57	51	50	17	101	81
NRG1_3p_2	16	58	3	20	39	49	21	18	28	15	55	9	15	11	34	8	47	43	18	24	4	65	68
NRG1_5p_1	34	165	8	44	159	199	59	24	128	12	174	65	32	11	174	29	70	89	15	135	5	255	79
NRG1_5p_2	24	163	13	55	127	151	37	31	109	15	120	43	26	18	126	21	47	65	28	113	4	200	39
NTRK1_3P1	14	10	7	18	8	11	15	11	7	10	3	11	8	16	8	9	15	8	10	16	4	18	19
NTRK1_3P2	16	10	5	35	14	20	15	16	6	8	4	9	15	6	18	5	18	16	9	15	2	23	24
NTRK1_3P3	10	10	4	23	13	18	5	13	10	9	4	10	12	10	12	4	13	18	4	13	3	18	10
NTRK1_3P4	22	9	3	40	16	15	9	21	16	5	7	7	21	13	13	8	22	11	8	22	3	13	23
NTRK1_5P1	6	7	6	15	7	7	7	9	9	7	3	3	8	8	9	6	8	12	13	8	7	6	18
NTRK1_5P2	3	3	1	8	3	8	3	3	1	1	2	3	4	3	2	1	1	3	3	7	2	12	10
NTRK1_5P3	13	8	6	18	11	25	8	16	4	6	8	7	11	10	4	4	14	9	13	8	1	15	19
NTRK1_5P4	20	20	17	37	25	28	20	26	19	17	16	27	38	19	20	18	19	21	22	16	16	20	24
Napsin A (NAPSA)	19	17	11	8217	27	29	93	6905	69	15	23	14	10	726	25	24	22	792	4023	5706	23	40	26814
PD1	8	8	7	14	36	30	26	21	21	47	16	14	10	45	17	6	66	20	40	29	5	17	15
PDL1	30	65	33	44	58	27	37	43	20	21	110	19	18	52	17	16	172	51	107	29	8	51	40
PRC1	30	648	124	145	562	476	87	99	45	63	107	31	27	89	26	188	106	144	176	48	9	451	168
RET_3P-1	15	679	5	17	15	16	24	18	24	7	5	8	17	7	20	10	23	28	8	34	4	27	24
RET_3P-2	16	709	8	25	27	16	34	38	44	6	9	25	36	28	32	22	46	37	21	42	16	43	45
RET_3P-3	18	1010	7	29	30	37	44	20	57	6	16	23	31	14	36	5	49	38	18	65	5	46	35
RET_3P-4	12	406	5	18	23	27	26	23	25	9	6	25	21	13	17	13	18	25	18	36	3	26	25
RET_5P-1	11	681	3	22	18	35	36	16	28	12	4	19	19	11	31	5	32	23	7	39	1	28	26
RET_5P-2	19	858	10	26	23	29	41	16	41	10	6	30	33	12	29	10	36	27	15	59	11	33	24
RET_5P-3	13	585	5	20	17	32	28	18	25	2	3	18	25	6	24	3	23	26	14	32	1	33	16
RET_5P-4	14	677	4	17	22	25	27	13	21	12	4	25	19	12	22	8	46	18	11	49	6	34	25
ROS1_3P-1	10	14	9	85	59	17	11	81	10	6	10	5	7	32	1	5	12	120	110	95	1	33	276
ROS1_3P-2	9	16	8	97	58	19	17	54	14	9	12	11	17	45	13	20	15	153	150	94	9	10	288
ROS1_3P-3	12	19	25	82	51	16	24	65	10	13	16	21	16	46	10	22	16	107	111	84	12	19	278
ROS1_3P-4	5	11	4	59	40	7	13	44	10	6	5	12	6	33	3	4	3	112	107	61	1	18	219
ROS1_5P-1	7	14	24	202	148	12	22	154	4	7	14	7	10	54	13	10	10	367	365	329	5	19	655
ROS1_5P-2	5	10	21	128	67	10	17	82	6	6	8	10	12	49	11	3	10	188	154	182	7	18	427
ROS1_5P-3	8	14	18	187	136	7	23	133	10	3	11	2	6	72	12	6	13	306	283	309	3	27	606
ROS1_5P-4	8	8	18	74	57	8	12	40	4	3	8	10	2	26	15	9	4	167	143	106	10	9	332
RRM2	30	2225	141	510	696	711	370	193	144	199	223	56	37	373	49	991	395	336	670	88	6	1239	276
SDC4-ROS1_S2:R32	12	18	12	24	25	29	17	15	20	10	7	8	16	7	7	5	19	23	16	19	4	27	13
SDC4-ROS1_S4:R32	10	9	5	7	9	17	11	8	7	3	8	7	11	8	6	16	14	3	9	6	7	11	6
SDC4-ROS1_S4:R34	15	24	18	22	29	27	28	25	15	23	20	29	18	26	29	23	17	17	22	19	11	16	46
SFTPB	8	21	9	3321	22	44	118	7685	46	7	6	8	11	1603	9	4	17	2001	3900	755	1	49	263
SFTPG	7	11	327	2668	12	8	573	767	17	9	10	12	16	53	29	8	9	77	418	366	11	17	4237
SLC34A2-ROS1_S13del2046:R32	1	3	6	6	8	6	14	8	9	4	1	9	4	8	5	13	11	10	5	7	9	6	5
SLC34A2-ROS1_S4:R32	18	40	17	27	111	120	48	21	22	6	16	10	22	23	15	18	29	33	61	15	7	83	22
SLC34A2-ROS1_S4:R34	7	15	5	13	12	9	7	7	8	7	1	15	8	10	7	6	14	13	9	6	3	13	8
SLC35A5	191	476	83	208	466	358	193	212	430	93	108	116	186	111	159	71	200	267	177	243	8	312	253
STMN2	29	296	54	13	24	19	17	38	21	16	11	14	41	22	13	25	16	9	15	40	7	16	14
SYP	46	1068	35	32	39	44	43	59	45	37	21	38	51	36	45	173	33	39	28	59	19	40	57
TFG-ALK_T5:A20	7	5	6	5	10	9	3	8	4	4	4	3	8	6	3	5	6	5	10	4	3	10	6
TFG-ALK_T5:A20	7	4	2	2	7	12	8	6	6	7	4	6	15	8	2	9	4	8	6	6	7	6	5
TFG-ALK_T6:A20	5	4	4	14	15	14	8	6	8	1	11	5	10	7	9	6	9	9	10	7	4	23	17
TP73L	180	730	23	624	5591	3800	585	116	1496	131	1569	425	236	194	1302	20	407	1265	266	1174	10	12254	264
TPM3-ROS1_T8:R35	8	17	5	10	13	14	9	6	8	4	5	9	13	5	8	7	8	10	14	2	7	18	10
TPSAB1	383	339	58	12437	2482	1998	1229	3447	2794	578	1089	2284	9420	2133	4843	6	4196	3758	109	5740	4	4273	1415
TREM1	40	237	302	188	103	103	20	903	34	24	319	29	34	67	59	14	124	303	633	36	1	66	828
TRIM14	217	358	121	448	568	321	251	394	515	377	238	176	177	317	200	44	514	563	341	343	18	526	341
TRIM24-BRAF_T5:B8	5	18	6	12	11	15	9	11	8	8	3	5	9	3	3	7	7	4	9	5	4	15	6
TRIM24-BRAF_T9:B9	9	10	8	10	8	10	10	4	13	8	3	10	11	5	5	9	12	12	12	7	9	8	9
TTC37	856	1194	130	597	799	642	520	698	765	422	217	303	437	313	486	293	515	694	446	532	14	887	814
TTF-1	115	328	61	137	190	221	118	144	208	133	123	77	97	81	108	77	115	221	158	180	6	217	231
TYMS	31	2417	86	454	331	1288	317	359	151	276	429	80	77	234	71	1380	424	476	616	193	5	969	646
UMPS	53	169	21	61	136	89	45	68	75	68	44	31	36	23	45	50	38	55	41	71	12	127	74
XPO1	686	1904	348	1103	2942	2103	788	952	1466	596	797	437	506	444	630	1144	899	1056	1047	965	7	2855	1800
ZMYND10	47	74	15	322	156	837	751	107	5966	151	78	834	375	13	484	10	132	766	197	444	11	29	247
ZNF236	153	382	49	138	210	270	145	214	206	191	85	79	128	77	136	174	158	231	117	169	3	271	251
ACTB	39414	127730	23367	82238	69252	73113	46276	91004	62246	60448	32409	33603	67894	51260	41319	7344	71833	131821	65533	66215	3	141775	119880
CLTC	1620	3754	533	2902	4065	2318	2300	2171	3530	881	1021	1118	1724	1119	1263	368	1683	3043	1403	2182	4	4445	5261
GAPDH	9176	17061	6091	15949	41426	55441	17349	10990	10206	4582	15224	3965	7427	7174	6971	6378	9450	12492	20591	7096	1	65185	14423
GUSB	378	598	209	477	629	492	453	545	563	177	171	187	297	332	305	101	443	462	252	574	3	467	946
HPRT1	355	937	125	757	1351	619	245	411	453	215	342	126	224	268	258	267	348	479	760	328	2	1136	678
MRPL19	494	580	153	689	1030	882	464	408	731	261	302	209	341	223	324	86	309	495	431	462	6	1017	730
OAZ1	3496	6681	1421	4252	6268	9219	3453	5596	8148	3700	4246	2629	3419	3782	3516	1316	4556	6084	4886	3939	7	8765	4952
PGK1	1691	3134	2191	6761	6987	4156	2165	4475	2138	1036	2870	687	987	1505	1281	810	2145	2730	5155	1875	3	8024	3580
POLR2A	707	2067	259	1155	1497	1510	1007	862	1696	653	482	568	788	575	717	356	1179	1276	507	1257	1	2738	1344
PSMC4	291	751	180	597	877	1196	447	446	973	224	293	275	298	253	286	136	431	495	472	391	7	1044	590
PUM1	635	1121	158	544	818	974	424	626	918	435	340	313	397	238	449	268	463	684	431	662	10	1170	817
RPLP0	9	9	9	6	20	16	6	10	8	5	5	10	7	7	10	15	18	6	13	6	8	14	5
SF3A1	491	796	192	593	1011	934	472	522	1168	505	431	380	508	274	532	93	571	659	330	689	1	1459	1145
TFRC	290	1693	503	1026	6650	4943	1616	1111	503	457	1984	160	294	649	380	364	753	700	1388	479	5	7440	806
TUBB	1204	8785	549	2469	3966	6632	1689	2645	1780	1623	1490	1031	1607	1716	1254	2061	2517	2987	1516	1789	4	3798	4531
NEG_A	1	3	1	3	6	4	1	4	1	4	1	4	4	1	1	3	3	3	3	3	3	5	4
NEG_B	6	10	1	4	3	6	3	6	1	3	4	1	4	2	2	4	5	2	7	3	4	12	8
NEG_C	4	6	5	6	12	8	4	5	3	4	1	4	4	5	6	6	7	5	10	6	7	7	12
NEG_D	6	4	1	3	5	2	2	11	1	1	2	1	2	4	2	2	5	5	5	3	1	1	4
NEG_E	1	6	1	3	4	6	3	5	1	1	2	3	6	1	3	3	5	4	5	4	3	6	6
NEG_F	6	22	3	19	29	21	15	19	8	11	4	13	17	7	10	3	16	21	23	7	6	49	20
POS_A	32414	27550	26044	26178	23810	26029	29891	31077	32292	27447	21092	26851	27824	22595	20996	28839	22230	24114	26780	22915	24354	25704	35742
POS_B	5299	4234	4000	4015	3665	4025	4703	4721	4944	4198	3557	4199	4431	3631	3349	4577	3815	3736	4153	3807	4019	4271	5494
POS_C	1416	1125	1174	1077	974	1089	1229	1292	1366	1120	1017	1136	1150	965	927	1256	1046	974	1079	992	1153	1425	1661
POS_D	197	164	172	209	160	149	202	208	211	156	134	175	187	159	148	204	174	163	167	155	168	186	234
POS_E	92	80	92	91	90	88	116	95	89	83	93	109	96	74	87	113	84	80	106	95	102	101	106
POS_F	44	30	26	41	26	27	32	41	33	31	18	25	35	32	22	37	35	28	39	34	25	29	44

Supplementary table 5. Raw data generated using NanoString gene expression. Number are presented as counts.
